# Public attitudes to genetic technology for invasive pest control and preferences for engagement and information: a segmentation analysis

**DOI:** 10.3389/fbioe.2024.1388512

**Published:** 2025-01-22

**Authors:** Elizabeth V. Hobman, Aditi Mankad, Lucy Carter, Kerry Collins

**Affiliations:** CSIRO Advanced Engineering Biology Future Science Platform, CSIRO Environment, Brisbane, QLD, Australia

**Keywords:** invasive pests, genetic technologies, segmentation, science communication, public perceptions

## Abstract

Advances in genetic technology hold promise in managing the increasing problem of invasive pests. The current study sought to improve our understanding of public perceptions, and potential public engagement pathways and information needs as the technology is researched and potentially developed for deployment. A survey of 1,149 Australians was conducted, and the sample was segmented into 4 groups based on their attitudes: Certain Objectors, Fence Sitters, Cautious Supporters, and Certain Supporters. ‘Light touch’ engagement activities appeared to satisfy most people; yet more intensive engagements could be appropriate for a small group who hold negative views towards the technology. Across the board, people wanted to know about the potential risks, and the regulation and controls surrounding the gene editing technology. Those who held more positive views also showed an interest in the scientific processes and techniques, while people who held more negative views wanted to know what was being done to deal with social and ethical issues. The results provide insight into 1) the diversity of views, and associated beliefs and feelings, among the public when confronted with a synthetic biology solution to an environmental problem, 2) how public engagement activities can be tailored to align with people’s engagement beliefs and stated preferences, and 3) what issues biotechnology developers should address as they endeavour to design genetic technology in a socially responsible way.

## 1 Introduction

Invasive animal and plant species are a major threat to native flora and fauna in Australia, listed as affecting 82% of threatened taxa in the country (Kearney et al., 2019). Among other factors such as disease, habitat loss and climate change, invasive species have been a dominant driver of nearly all extinctions for the past 60 years (Woinarski et al., 2019). Invasive pests also have substantial negative impacts on the agricultural industry through both production losses and expenditure on pest management (McLeod, 2016). Overall, the total cost of invasive species to the Australian economy is in the order of $25 billion a year (Bradshaw et al., 2021).

Conventional pest control techniques include integrated chemical and physical management practices (e.g., poison baiting with fencing), direct intervention (e.g., trapping), and biological control. While these pest control strategies have achieved some success, they are labour- and cost-intensive, which is a significant impediment to large-scale deployments (Glen et al., 2013). Some pest management strategies also raise animal welfare concerns and have unintended ecological consequences (Messing and Wright, 2006). The problems associated with current pest control strategies have encouraged scientists to explore alternative approaches (Moro et al., 2018). Emerging solutions from the field of synthetic biology could potentially control invasive species in a way that is more scalable, targeted, and cost-effective, and that reduces animal welfare concerns (Piaggio et al., 2017; Segelbacher et al., 2021; Teem et al., 2020). One synthetic biology approach involves deleting a target gene and/or inserting an engineered gene (or set of genes) to distort sex ratios in offspring. For example, the ‘daughterless’ approach involves genetically modifying males to carry transgenes so that they either do not produce daughters or so that XX offspring (normally female) are induced to develop instead as sterile males, while XY offspring develop as normal, fertile males. Paired with a gene drive to bias inheritance of the modified gene through sexual reproduction, this approach could eventually result in population decline through reduced fecundity (Piaggio et al., 2017; Teem et al., 2020).

Laboratory experimentation with genetic technologies are yielding promising results in insects and mice models (Birand et al., 2022) and further investigations are underway to evaluate their effectiveness, including unintended effects (Price et al., 2020; Teem et al., 2020). In addition to these technical and ecological risk assessments, it is also important to undertake social science studies that explore the interrelationships between people in society and the proposed technological solutions. These societal assessments are imperative since people will be the ones who ultimately accept or oppose the deployment of the technology (Campbell et al., 2015; Peltzer et al., 2019). When armed with an understanding of societal concerns, needs, and wants, researchers are better positioned to design and deliver a technological innovation that will be accepted and adopted by society at large. Public engagement processes have been promoted as an essential component of pest control initiatives (Peltzer et al., 2019), especially in the context of genetic technologies where the outcomes of such engagements are considered just as crucial as scientific results (National Academies of Sciences Engineering and Medicine, 2016; Thompson, 2018). Prior research in Australia has also shown that the public attach great importance on citizens being kept informed about new scientific advances in pest control (including, but not limited to genetic control solutions) (Fisher et al., 2013). Accordingly, jurisdictions throughout the world have recognised the need for public engagement in the context of developing and using genetic technologies for environmental conservation purposes (Convention on Biological Diversity, 2018; National Academies of Sciences Engineering and Medicine, 2016). The need for public engagement in research pertaining to genetic technologies is consistent with the broader call for societal engagement in research and innovation processes (Bauer et al., 2021).

But what exactly is meant by ‘public engagement’? Public engagement encompasses all kinds of activities that aim to bring a public perspective into the development of an emerging technology (Rerimassie, 2016). These activities might seek to engage people in a two-way dialogue, conversation or flow of information, though one-way communication which informs or educates the public may also be classified as a public engagement activity (Rerimassie, 2016). In the context of synthetic biology, several public engagement activities have already been performed, including a range of dialogue-based activities such as citizen panels/juries, stakeholder workshops, consensus conferences, focus groups, deliberative polling and cafes scientifiques (mainly undertaken in Europe) (Ancillotti et al., 2016; Bauer and Bogner, 2020; Bhattachary et al., 2010; Bubela et al., 2012; Navid and Einsiedel, 2012; Pansera et al., 2020; Rerimassie, 2016; Van Mil et al., 2017). Additionally, qualitative and quantitative social research has been performed to assess public perceptions of synthetic biology (Akin et al., 2017; Cormick and Mercer, 2019; Pauwels, 2013), including in the specific case of genetic technologies for pest control (Fisher et al., 2013; Jones et al., 2019; Kohl et al., 2019; 2020; MacDonald, Edwards, et al., 2021; MacDonald et al., 2020; MacDonald, Neff, et al., 2021). Although quantitative surveys do not allow for dialogue, they still facilitate a two-way flow of information and have the potential to elicit information on public values, attitudes and preferences for incorporation into research and science policy discussions and decision-making (Sturgis, 2014).

Despite a general consensus that public engagement is needed when it comes to introducing genetic technologies, there is less shared understanding of the means and processes of public engagement that are required to achieve meaningful and impactful engagement (Bauer et al., 2021; Grogan, 2014; Seitz, 2016; Stilgoe et al., 2014; Stirling et al., 2018). Some of the practical questions that need to be addressed include: Who will participate in the engagement, and how will they be recruited? What will be the content and process of engagement? Where and when should engagement occur along the technology development-implementation pathway? and Who will be responsible for facilitating the engagement? The answers to these questions may be largely directed by the purpose/s of engagement, of which there are many. Engagement can be motivated by ‘substantive’ reasons–to achieve better decisions through the co-production of knowledge with the public, ‘normative’ reasons – to demonstrate that appropriate democratic procedures were followed to uphold equity and justice concerns, and/or by ‘instrumental’ reasons – to achieve the organisers” goals (e.g., raise knowledge, build trust, gain acceptance) (Bauer et al., 2021; Seitz, 2016; Stirling et al., 2018). In line with these different purposes or reasons, the nature of public engagement can vary along a continuum, from being more explorative, reflective, and problem-focussed (known as the ‘opening up’ approach), to being more non-negotiable, pre-determined, and solution-focussed (known as the ‘closing down’ approach) (Russell et al., 2022). Between these two extremes, public engagement may also be designed to respond pragmatically to public concerns/views by creating acceptable terms or conditions under which the solution is deployed (known as the ‘leaving ajar’ approach) (Russell et al., 2022).

When deciding on the best engagement approach to suit one’s needs, it is also important to recognise the public themselves will hold their own views on the extent to which they should be involved in making decisions about the science/technology. For instance, the Special Eurobarometer survey undertaken in 2021 revealed most people (i.e., 84%) across 27 European Union member states believed the public should either be informed or consulted with (and their opinions seriously considered) – while a small proportion thought that public opinion should be the main concern when making decisions about science and technology (8%), or alternatively that the public does not need to be involved at all (7%) (European Commission, 2021). Thus, another critical question to be addressed when designing public engagement is: How do different publics want to be engaged? Even though participatory dialogue is perceived by many as the ‘gold standard’ of democratic governance, there is evidence to suggest that the public may not be so keen. Research has revealed only a small, self-selected fraction of the population, usually of high socio-economic status and with high interest and strong views on the topic, are willing to participate in such activities (Sturgis, 2014). Indeed, it has been widely accepted that people selectively form around threatening technoscientific objects and matters of concern to them, and it is these ‘mini-publics’ or ‘issue-oriented publics’ who are more likely to engage in participatory dialogue activities (Jasanoff, 2014). While these publics are usually knowledgeable, it is possible that their views and recommendations may differ from the wider population, resulting in non-representative findings (Sturgis, 2014). Considering this problem, researchers have suggested asking the public about how they would like to be involved in decisions about science (Sturgis, 2014), and broadening the scope of engagement platforms and spaces (e.g., online social media and open science platforms) (Stilgoe et al., 2014).

A potential source of information that already exists, which may guide the development of a public engagement process is social science research on public values, attitudes and support for the use of genetic technologies for pest control. While communication or engagement preferences may not always be specifically asked of survey participants, this research can still provide valuable insights into the variability or conditionality in people’s receptiveness towards new technology, the critical concerns they have and may like to discuss, and the sources of information they trust. Information along these lines may be used to inform the content of public engagement activities, and delivery pathways. The few empirical studies that have specifically explored public perceptions of genetic technology for pest control (Black et al., 2021; Jones et al., 2019; Kohl et al., 2019; 2020; MacDonald, Edwards, et al., 2021; MacDonald et al., 2020; MacDonald, Neff, et al., 2021) have revealed considerable heterogeneity or variation in public support or acceptability with affirmative scores ranging from around 20%–60%, and mean scores hovering around the mid-point on the relevant Likert scale. Support appears to be heavily conditional on the genetic technique or method used and how the problem is framed^1^, but also on the variability in attitudes, beliefs, and worldviews across individuals^2^. Overall, such research shows that while many people are moderately supportive of genetic technology for pest control, there is still a sizeable proportion who are either opposed or undecided. Importantly, regardless of support, people across the board raised concerns about the possible risks and potential for dual use (i.e., malicious improper use) and misuse (i.e., unintentional and accidental improper use); negative effects on humans, animals, and the environment; the technology’s efficacy (i.e., scepticism regarding its benefits), regulation, and cost-effectiveness (Jones et al., 2019; Kohl et al., 2020; MacDonald, Edwards, et al., 2021; MacDonald et al., 2020; MacDonald, Neff, et al., 2021).Such concerns may therefore be universal matters to be included in public engagement initiatives as standard. However, since research shows there is considerable diversity in general support across the population, driven by different underlying psychological values, attitudes, and beliefs, it is anticipated that a tailored approach will be required when engaging with the public.

In summary, it is unequivocal that public engagement is essential when considering the development of genetic technology for invasive pest control, yet questions remain on how such engagements are best accomplished. It can be anticipated that diverse engagement methods will be required to meet the needs of different organisers, stakeholders, and audiences. Indeed, social science studies suggest that a tailored approach may be required to account for the heterogeneity in attitudes towards genetic technology for pest control, observed across the population. Spending the time to adequately plan and design fit-for-purpose engagement activities is important given that public engagement exercises can be very costly and time-intensive (Grogan, 2014), and can heavily influence broader public debate, trust, and the future public image of the technology under examination (Seitz, 2016). To this end, the current study sought to commence some of this preparatory groundwork by exploring the information needs and engagement beliefs and preferences of different segments within the general public when it comes to learning more and being involved in decisions about genetic technologies for invasive pest management. To our knowledge, this is the first foray into asking the public about how they would like to be involved, engaged, and/or informed. We believe this as a fundamental and essential first step in designing public engagement initiatives that will meet the preferences of the wider population – not just those who are personally interested or have a personal stake in the issue. Only one New Zealand-based study has previously divided the population into segments with different perceptions of gene technology for invasive pest control (MacDonald, Edwards, et al., 2021; MacDonald et al., 2020). We first posed the following research question to identify the presence of different attitudinal segments in the population:

Research Question 1 Are there discernible segments in the population that differ in their attitudes towards the development of genetic technology for invasive pest control?

After dividing our study sample into segments, we then sought to address the following research question to determine what public engagement approaches and communication strategies might be needed.

Research Question 2 What are the public engagement preferences, beliefs, and information needs for different segments?

### 1.1 Relevant factors for segmentation

While recognising there are broader factors in the socio-political, cultural context, and information climate that affect how people perceive science, for the purposes of our study we focussed explicitly on measuring individual characteristics known to influence public perceptions of science (Wirz et al., 2020). Following the precedent set by previous science communication researchers who have performed segmentation analyses to understand the attitudes of different segments in the population (Füchslin, 2019; Schafer et al., 2018), we selected multiple individual variables that together provide a holistic understanding of public perception towards the use of genetic technologies for invasive pest control. In the absence of a clear theoretical framework to guide variable selection for the segmentation analysis, we drew on prior empirical studies examining public attitudes to synthetic biology and genetic technologies in general, not just those confined to the pest control space. These empirical studies provide evidence to show which individual variables are important to people when considering new genetic technologies. For instance, prior research has shown that, when evaluating synthetic biology or related technologies and their applications, the general public consider the perceived/expected benefits or relative advantage of the technology over other solutions (Akin et al., 2017; Brossard and Nisbet, 2006; Fisher et al., 2013; Jin et al., 2019; Poortinga and Pidgeon, 2006), the perceived/unknown risks (including secondary/dual uses and misuses) (Akin et al., 2017; Brossard and Nisbet, 2006; Fisher et al., 2013; Jin et al., 2019; Jones et al., 2019; Poortinga and Pidgeon, 2006), how they feel about the technology (Poortinga and Pidgeon, 2006), what they know about the solution (Brossard and Nisbet, 2006), and whether they trust the actors responsible for developing, making decisions about, or properly regulating, the solution (Akin et al., 2017; Brossard and Nisbet, 2006; Kohl et al., 2020; Poortinga and Pidgeon, 2006). Other contributing factors to people’s decision-making include underlying values such as their pro-environmental beliefs (MacDonald et al., 2020), and their global attitude or evaluative assessment of the technology (Poortinga and Pidgeon, 2006). While these variables might be considered heterogeneous, they are the multidimensional factors that reflect different components of a person’s overall attitude. That is, attitudes as originally conceived in the social psychological literature (Azjen, 1989), includes cognitive (one’s beliefs), affective (one’s feelings), and conative (one’s behavioural intention) elements. Thus, all these elements were important to provide a holistic assessment of a person’s overall attitude towards genetic technologies for invasive pest control.

The current study was undertaken in Australia, a country which has experienced the introduction and establishment of many invasive pest species since European settlement. In recognition of the threats that such pests pose to the nation’s biodiversity and agricultural industries, Australia has developed a co-ordinated national approach to managing the threat of pests (Invasive Plants and Animals Committee, 2016), including the creation of the government-funded Centre for Invasive Species Solutions (CISS) – a collaborative research, innovation and engagement organisation tasked with tackling the problem of invasive pests and weeds (Sheppard and Glanznig, 2021). CISS has suggested genetic technologies for animal pest control are a potentially cost-effective, easy, quick, scalable solution for the 21st century (Sheppard and Glanznig, 2021). Australia is also a country that has a well-established framework for regulating gene technology with various GMOs receiving approval for contained research work, or for commercial or limited/controlled release into the environment (see https://www.ogtr.gov.au). Proactive reviews and reforms continue to be performed to ensure its regulatory processes stay current with advancements in technology. For instance, a guidance document has been recently drafted to assist researchers in navigating the regulatory requirements for environmental release of organisms containing a gene drive (Commonwealth of Australia, 2023). Given the invasive pest problem context and the nation’s proactive regulatory climate concerning GMOs, Australia presents a suitable case when considering public attitudes to genetic technologies in the pest control space.

## 2 Materials and methods

A detailed description of methods is provided in Supplemental Method. A cross-sectional online survey of 1,149 Australians was conducted in late 2018^3^. This sample size was chosen based on prior research, which suggests a sample size of at least 30 times but ideally 70 times the number of variables is adequate for reliable and valid segmentation results (Dolnicar et al., 2014; 2016). Cross-cutting quotas were established to ensure the sample was representative of the national population in age (≥18 years) and gender; a range of educational levels were also represented. Participants were recruited via an external third-party online research provider with each participant receiving a token incentive for survey completion. At the start of the survey, participants were provided with a general definition of synthetic biology. Participants then viewed a slideshow presentation (available at https://research.csiro.au/synthetic-biology-fsp/public-attitudes/) that explained the problem of invasive pest species in Australia, current methods of control, and a possible synthetic biology solution to help manage pests. The animal pests that were listed included: feral rabbits, pigs, cats, dogs, carp, and cane toads. Furthermore, two sex-biasing examples were provided: 1) genes of feral cats could be modified so that all offspring are a single sex, reducing opportunities to mate, and 2) genes of European carp could be modified so that females only produce infertile males.

To minimise the length of the survey and reduce respondent fatigue, single item measures were utilised for some variables. The use of single-item measures is considered appropriate for constructs that are unidimensional and concrete (Bergkvist and Rossiter, 2007).

### 2.1 Public engagement preferences and information needs

Belief in appropriate public involvement in decision-making was measured by asking participants to select what level of public involvement they feel is appropriate, when it comes to making decisions about the technology (adapted from European Commission, 2012; 2021). Four options were presented, representing increasing levels of involvement (ranging from ‘the public does not need to be involved’ to ‘the public should be directly involved’), along with a ‘don’t know’ option (Mean = 2.65, SD = 0.73) (see note to Table 3 for the complete wording for all response options).

Importance of having a say in how the technology is developed and implemented was measured with 2 questions and responses combined (Cronbach’s alpha = 0.91) (Mean = 3.10, SD = 1.11). These questions were drawn from the organisational justice literature, which has highlighted the importance of individual voice in decision-making (Price et al., 2006). ‘Having a say’ in decision-making is a component of procedural justice and has been identified as important in the acceptance of technology (Devine-Wright, 2013).

Engagement preferences were measured by asking participants the extent to which they would want to be involved in 4 different public engagement activities, as listed in Table 3 (1 = not at all to 5 = very much so). These activities were chosen to represent some of the most common options for community engagement, including activities that aimed to inform (i.e., receive results of research – e.g., a summary report), consult (i.e., access information and/or provide feedback through social media), and involve (i.e., formally contribute to making decisions – e.g., written submissions to the relevant authority; participate in public information sessions – e.g., town hall meetings) people in decision-making.

**FIGURE 1:**
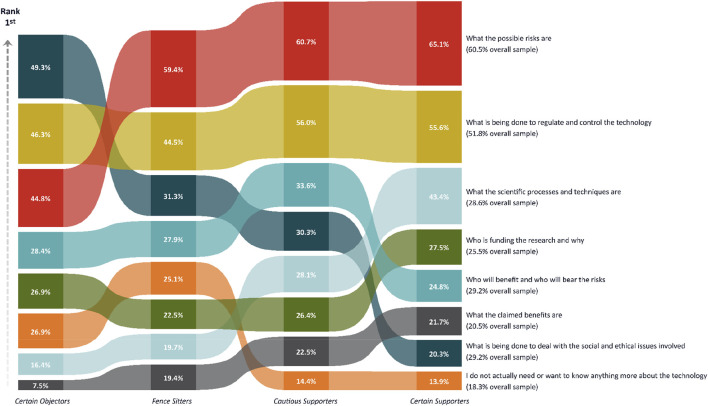
The percentage (%) of respondents in each class nominating a particular information topic as their “top 3 information needs”.

Personal information needs were measured by asking participants to select the three top issues that they would like to know more about (1 = being the most important). These issues are presented in Figure 1. These information needs were chosen to represent some of the more common issues raised in previous qualitative research on synthetic biology (Bhattachary et al., 2010; Pauwels, 2013).

### 2.2 Segmentation variables

Segmentation was based on the following variables, which are grouped together based on their overarching construct. The means, standard deviations and intercorrelations among these variables is in Supplemental Table S1.

#### 2.2.1 Pro-environmental attitude

Pro-environmental attitude was measured using 9 items from the 15-item revised New Ecological Paradigm (Dunlap et al., 2000). The items were coded so that high scores reflected a pro-environmental attitude. All items loaded on a single factor, with acceptable internal consistency (Cronbach’s alpha = 0.76).

#### 2.2.2 Perceptions of the pest problem

Perceptions of the invasive pest problem was measured by asking people to rate their awareness of the pest problem in Australia (1 = no awareness, 3 = medium awareness, 5 = high awareness), and how problematic they thought the pest problem was (1 = not a problem at all to 5 = a very big problem). These questions were developed from an understanding of the protection motivation literature; the theory explains how fear appeals and coping processes influence attitude change (Rogers, 1975; 1983).

#### 2.2.3 Understanding of the genetic technology

Understanding of the genetic technology was measured through a comprehension test of the information contained in the slideshow. Participants answered three true or false questions, two of which were true: and one which was false. Correct answers were summed with scores ranging from 0 to 3. Subjective understanding was also measured by asking participants to report how well they felt they understood what the technology was about (1 = not very well, 3 = moderately well, 5 = very well).

#### 2.2.4 Attitudes towards the technology

Drawing on literature examining the nature of attitudes (Ajzen, 1991; 2008), participants were asked to provide an overall favourable or unfavourable evaluation of the genetic technology through a consideration of its outcomes (i.e., I feel this technology would be: 1 = harmful … 5 = beneficial; 1 = bad … 5 = good; and 1 = risky … 5 = safe’) (termed ‘evaluative attitudes’). Scores were combined to provide an assessment of global attitude towards the gene drive solution (Cronbach’s alpha = 0.88). Participants were also asked to evaluate the genetic technology by considering its ethics and morality (i.e., I feel this technology would be: 1 = unethical … 5 = ethical; and 1 = immoral … 5 = moral’) (termed ‘value-based attitudes).

In addition to assessing attitudes, we also measured the extent to which participants felt undecided or ambivalent in their attitudes about the technology (1 = not at all to 5 = very much) (Priester and Petty, 1996).

#### 2.2.5 Perceived effectiveness

Drawing again on protection motivation theory (Rogers, 1975; 1983), participants were asked to indicate the extent to which they thought the technology would help reduce or eliminate invasive pests (termed ‘response efficacy’ since it concerns the effectiveness or expectancy that the technology as a ‘coping response’ might avert the problem) (1 = would not help at all to 5 = would be very helpful). Referring to the diffusion of innovation theory (Rogers, 2003), one key characteristic that influences the adoption process is ‘relative advantage’ – this component was measured by asking participants whether they thought the technology would be better than current methods of invasive pest management (1 = strongly disagree to 5 = strongly agree).

#### 2.2.6 Concerns regarding improper use and long-term impacts

In the absence of established questions, we developed questions to assess degree of concern about the potential for improper use and long-term impacts of the technology. First, concern regarding the potential for improper use was measured by asking participants to what extent they were concerned that the technology would get into the wrong hands and be used for bad purposes (i.e., dual use), and that the technology could be inadvertently misused, leading to unintended negative consequences (i.e., misuse) (1 = not concerned to 5 = extremely concerned). These items were highly correlated (*r* = 0.79) and combined to provide a measure of concern regarding improper use. Second, concern regarding the long-term impacts was measured in the same manner with three statements about the impacts on humans/animals, the natural environment and whether the consequences can be effectively controlled (1 = not concerned, 3 = moderately concerned, 5 = extremely concerned). The three items were combined to provide a measure of long-term impacts (Cronbach’s alpha = 0.89).

#### 2.2.7 Emotions

Affect, both positive and negative, were measured by asking participants about how they felt when reading through the information about the technology (1 = not at all to 5 = very much) (adapted from the Positive and Negative Affect Schedule: (Watson et al., 1988). The ratings for hopeful, excited, and curious were combined to reflect positive affect (Cronbach’s alpha = 0.80), and the ratings for concerned, afraid, and angry were combined to reflect negative affect (Cronbach’s alpha = 0.80).

#### 2.2.8 Support

Support for the development of the gene drive technology was measured by asking participants the extent to which they would support the development of the technology (1 = would not support to 5 = would strongly support).

#### 2.2.9 Trust and confidence in governance

Drawing on research examining the role of social trust in those who manage hazards (Siegrist and Cvetkovich, 2000), trust in scientists was measured by asking ‘How much do you trust that scientists working on this technology would develop it responsibly?’ (1 = no trust, 3 = moderate trust, 5 = high trust) and trust in government was measured by asking ‘How much do you trust the government agency that would be responsible for approving and regulating this technology–for example, the Office of the Gene Technology Regulator?’ (1 = no trust, 3 = moderate trust, 5 = high trust). Confidence in regulation was measured by asking participants to rate their level of agreement (1 = strongly disagree to 5 = strongly agree) with two statements (adapted from Zhang et al. (2018) (Cronbach’s alpha = 0.87) (Van Mil et al., 2017).

### 2.3 Data analysis: segment identification

Latent profile analysis is a person-centred statistical method for identifying related cases from multivariate continuous data (Howard and Hoffman, 2017; Spurk et al., 2020; Woo et al., 2018). Six latent profile models were requested using STATA/MP 17 (StataCorp, 2021) and a 4-class solution was selected based on improvements in Bayesian Information Criterion (BIC), the conceptual interpretability and utility of the class structure, and adequacy of class sizes. The 4-class profile solution resulted in classes that were distinct and interpretable, with all participants allocated to a class. A follow-up linear discriminant analysis resulted in over 93% of participants being classified to the correct class (i.e., 95.5% for Class 1, 95.5% for Class 2, 93.8% for Class 3% and 97.6% for Class 4). The mean scores for the 4 classes are presented in Table 1. A description of the 4 classes is presented in Table 2 (the full set of analysis associated with class comparisons is detailed in Supplemental Results and accompanying Supplemental Tables S1–3).

**TABLE 1:** Group comparisons across the 4 classes (score range: 1-5, where higher scores indicate stronger alignment with variable).

Indicator	Response scale range	Overall sample mean	Class 1 *Certain Objectors*	Class 2 *Fence Sitters*	Class 3 *Cautious Supporters*	Class 4 *Certain supporters*	Significance test (ANOVA)
		*n* = 1149	*n* = 67 (5.83%)	*n* = 355 (30.90%)	*n* = 432 (37.60%)	*n* = 295 (25.67%)	
Pro-environmental attitude	1–5	3.68	3.90	3.64	3.67	3.69	F(3,1145) = 4.08, *p* = 0.007
Problem awareness	1–5	3.91	3.87	3.29	4.01	4.51	F(3,1145) = 75.95, *p* = 0.000
Threat severity	1–5	4.10	3.42	3.50	4.28	4.72	F(3,1145) = 136.37, *p* = 0.000
Comprehension	0–3	2.55	2.40	2.19	2.66	2.86	F(3,1145) = 47.07, *p* = 0.000
Subjective understanding	1–5	3.23	3.37	2.69	3.19	3.89	F(3,1145) = 115.75, *p* = 0.000
Attitude – evaluative	1–5	3.54	1.40	2.84	3.73	4.58	F(3,1145) = 899.28, *p* = 0.000
Attitude – value-based	1–5	3.45	1.37	2.71	3.63	4.54	F(3,1145) = 649.95, *p* = 0.000
Attitude - undecided	1–5	2.61	1.97	3.38	2.75	1.51	F(3,1145) = 243.22, *p* = 0.000
Response efficacy	1–5	3.90	2.55	3.23	4.05	4.79	F(3,1145) = 387.10, *p* = 0.000
Relative advantage	1–5	3.80	2.04	3.19	3.95	4.72	F(3,1145) = 443.81, *p* = 0.000
Concern – improper use	1–5	3.38	4.61	3.69	3.40	2.68	F(3,1145) = 118.92, *p* = 0.000
Concern – long-term impacts	1–5	3.28	4.69	3.65	3.32	2.47	F(3,1145) = 206.61, *p* = 0.000
Positive affect	1–5	3.39	1.91	2.75	3.61	4.16	F(3,1145) = 368.12, *p* = 0.000
Negative affect	1–5	2.29	3.85	2.72	2.17	1.61	F(3,1145) = 214.37, *p* = 0.000
Support for development	1–5	3.67	1.40	2.87	3.96	4.74	F(3,1145) = 923.39, *p* = 0.000
Trust in scientists	1–5	3.47	1.70	2.94	3.63	4.28	F(3,1145) = 325.15, *p* = 0.000
Trust in government	1–5	3.07	1.40	2.61	3.19	3.84	F(3,1145) = 215.44, *p* = 0.000
Confidence in regulation	1–5	3.33	1.66	2.82	3.42	4.19	F(3,1145) = 323.13, *p* = 0.000

**TABLE 2:** Summary of results for the 4 classes.

Classes	Demographics	Engagement preferences and information needs
*Certain Objectors* (n = 67, 5.83%) held the most negative evaluative and value-based attitudes and feelings towards the solution. While they held the most pro-environmental attitudes and were highly aware of the pest problem, they did not consider invasive pests as very problematic. They were the least supportive of the solution, rating its effectiveness and relative advantage over current methods lower than all other classes. They were the least trusting of scientists and the government who would be responsible for approving and regulating the solution and held the greatest concerns about improper use, and long-term impacts. They felt less undecided about the technology	• Female-dominated (68.7%) • Younger-to-middle aged representation (77.6% aged between 18 and 54)	• Strong interest in having a say • Strong interest in lighter touch engagement activities, however, they also showed a reasonably strong interest in more intensive engagement • More interested in knowing about what is being done to deal with social and ethical issues, the regulation and controls surrounding technology, and the possible risks
*Fence Sitters* (n = 355, 30.90%) held neutral evaluative and value-based attitudes and feelings towards the solution. While they held moderate-to-high pro-environmental attitudes, they were less aware of the pest problem and did not consider it as a very serious problem. They reported the lowest understanding of the solution, which was also reflected in their objective comprehension scores. They were moderately supportive of the solution, rating it as moderately effective, and somewhat better than other pest control methods. They expressed some concerns about improper use, and long-term impacts and expressed the most undecidedness compared to all other classes. They held moderate trust in scientists and government and were reasonably confident in the regulation of the solution	• Slightly female-dominated (60.6%) • Younger-to-middle aged representation (80.8% aged between 18 and 54)	• Moderate interest in having a say • Moderate interest in lighter touch engagement activities. Low interest in more intensive engagement • More interested in knowing about possible risks and the regulation and controls surrounding technology
*Cautious Supporters* (n = 432, 37.60%) held more favourable evaluative and value-based attitudes and feelings towards the solution. They held moderate-to-high pro-environmental attitudes and were quite aware of the pest problem, rating it as a big problem. They also reported moderate understanding of the solution. They were very supportive of the solution, rating it as highly effective and better than current methods. They were still moderately concerned about improper use, and long-term impacts and reported higher levels of undecidedness. They exhibited moderate to strong trust in scientists and government and were more confident in the regulation of the solution	• Slightly female-dominated (54.6%) • All ages representative (43.9% aged between 18 and 44; 56.1% aged 45 and over)	• Moderate interest in having a say • Moderate interest in lighter touch engagement activities. Low interest in more intensive engagement • More interested in knowing about possible risks and the regulation and controls surrounding technology
*Certain Supporters* (n = 295, 25.67%) held the most favourable evaluative and value-based attitudes and feelings towards the solution. They held positive pro-environmental attitudes and the greatest awareness of the pest problem, rating it as a very big problem. Their knowledge and understanding of the solution were relatively strong. They showed strong support for the solution, believing that it would be highly effective and better than current methods. While they showed some concern regarding improper use, and long-term impacts, these concerns were less than that observed for other classes. They were also the least undecided about the solution. They expressed strong trust in scientists and government and were very confident in the regulation surrounding the solution	• Slightly male-dominated (56.3%) • Middle-to-older aged representation (68.8% aged 45 and over)	• Moderate-to-low interest in having a say • Moderate interest in lighter touch engagement activities. Low interest in more intensive engagement • More interested in knowing about possible risks, the regulation and controls surrounding technology, and the scientific processes and techniques involved

## 3 Results

Table 3 presents the public engagement preferences and beliefs overall, and across the different segments. Belief in the appropriate level of public involvement was moderate overall, with most people believing the public should be consulted with and their opinions considered (42%) or that the public should be kept informed (∼38%). Fewer people thought the public should be directly involved (∼11%), or not involved at all (∼3%). Significant class differences were observed where Certain Objectors believed the public should be more involved compared to all other classes. In fact, almost half (48%) of Certain Objectors thought the public should be directly involved, while only between ∼7% and 13% of the other classes thought the same. Many *Certain Objectors* also favoured the public being consulted with, and their opinions considered (∼36%). This type of consultative engagement was also supported by many Fence Sitters (∼49%) and Cautious Supporters (∼47%), and Certain Supporters (∼30%) – though with regard to the latter class, more people believed the public should simply be kept informed (∼56%). Class differences also were observed between Fence Sitters, Cautious Supporters, and Certain Supporters–with each class in turn reporting lower levels of public involvement.

**TABLE 3:** Group comparisons of means and percentage (%) scoring above the mid-point, on belief in appropriate public involvement, and personal preferences for participation in publice engagement activities across the 4 classes.

	Response scale range	Overall sample *n* = 1149 (100%)	Class 1 *Certain Objectors n* = 67 (5.83%)	Class 2 *Fence Sitters n* = 355 (30.90%)	Class 3 *Cautious supporters n* = 432 (37.60%)	Class 4 *Certain supporters n* = 295 (25.67%)	Significance test of means (ANOVA)
Which of the following most accurately reflects your feelings about the appropriate level of public involvement when it comes to making decisions about this technology?	1–4^a^	2.65	3.38	2.85	2.59	2.37	F (3,1075) = 49.40, *p* = 0.000
		> mid-point 53.3%	> mid-point 83.6%	> mid-point 61.1%	> mid-point 53.5%	> mid-point 36.6%	
How important would it be for you to have a say in how this technology is [developed/implemented]?	1–5^b^	3.10	4.04	3.23	3.08	2.77	F (3,1145) = 28.40, *p* = 0.000
		> mid-point 41.2%	> mid-point 74.6%	> mid-point 41.4%	> mid-point 40.3%	> mid-point 34.6%	
I would want to participate in public information sessions	1–5^c^	2.68	3.37	2.78	2.65	2.45	F (3,1145) = 11.83, *p* = 0.000
		> mid-point 23.3%	> mid-point 47.8%	> mid-point 19.7%	> mid-point 24.3%	> mid-point 20.7%	
I would want to access information/provide feedback through social media	1–5^c^	3.10	3.97	3.04	3.11	2.96	F (3,1145) = 12.94, *p* = 0.000
		> mid-point 39.9%	> mid-point 70.2%	> mid-point 33.8%	> mid-point 41.0%	> mid-point 38.6%	
I would want to formally contribute to decisions (e.g., written submissions)	1–5^c^	2.48	3.28	2.68	2.42	2.16	F (3,1145) = 20.53, *p* = 0.000
		> mid-point 20.9%	> mid-point 50.8%	> mid-point 20.6%	> mid-point 18.5%	> mid-point 18.0%	
I would want to receive results of research (e.g., summary report)	1–5^c^	3.17	3.85	3.06	3.15	3.20	F (3,1145) = 8.10, *p* = 0.000
		> mid-point 41.2%	> mid-point 67.2%	> mid-point 33.8%	> mid-point 40.0%	> mid-point 45.8%	

Notes.

^a^ 1 = public does not need to be involved in decisions about this technology, 2 = public should be kept informed of decisions made about this technology, 3 = public should be consulted with and opinions considered, when making decisions about this technology, 4 = public should be directly involved in making decisions about this technology, 9 = don’t know (not included in the calculation of the mean or statistical analysis).

^b^ 1 = not important, 5 = very important.

^c^ 1 = not at all, 5 = very much so.

Importance of having a say in how the technology is developed and implemented was moderate overall–roughly 40% indicated that this would be important to them. Significant class differences were observed where the majority of Certain Objectors (∼75%) placed importance on having a say, while less than half of respondents in the other classes did so. In fact, only around 35% of Certain Supporters indicated that having a say was important to them. In terms of how respondents preferred to be engaged, overall, participants were moderately interested in the ‘light touch’ passive engagement activities of receiving results of research and accessing information and/or providing feedback through social media (∼40% indicated interest). There was slightly less interest in formally contributing to decisions and participating in public information sessions (∼20% indicated interest). Significant class differences were observed again in that Certain Objectors expressed a greater interest–in the order of 20–30 percentage points higher–in all forms of engagement, as compared to all other classes.

Personal information needs are visually represented in Figure 1. Across the entire sample, most participants prioritised information concerning the possible risks of the proposed technology (n = 695, 61%) and what was being done to regulate and control the technology (n = 595, 52%). Conversely, a small percentage (n = 235, 20%) indicated that they wanted to know what the claimed benefits were and others who did not need or want to know anything more about the technology (n = 210, 18%). This pattern of results suggests that most people may be more focussed on risks and risk management. Yet, there were several statistically significant differences observed between the classes in the types of information nominated. Some of the more notable differences were that Certain Objectors were far more interested in receiving information about ‘what is being done to deal with the social and ethical issues,’ as compared to all other classes. They were also much less interested in receiving information about the ‘claimed benefits’ and somewhat less interested in knowing about the ‘possible risks,’ as compared to all other classes. Additionally, *Certain Supporters* reported greater interest in information about ‘scientific processes and techniques’ compared to all other classes.

## 4 Discussion

The importance of engaging with the public to inform the development of new technologies such as gene editing invasive animal pest species is unequivocal, yet questions remain about how this objective might best be accomplished (Sturgis, 2014). To guide these efforts, the current study is–to our knowledge–one of the first to seek to understand the general public’s information needs, interest in having a say, and beliefs and preferences for participating in different types of engagements when considering the specific case of gene editing for pest control.

Before delving into the results relevant to public engagement, we first discuss how our results compare with previous public perception studies conducted in New Zealand (Black et al., 2021; MacDonald, Edwards, et al., 2021; MacDonald et al., 2020; MacDonald, Neff, et al., 2021) and the U.S. (Jones et al., 2019; Kohl et al., 2019; 2020). While precise and valid comparisons cannot be accomplished–due to fundamental differences in research methods (such as the description of the genetic technology and how such information was presented to participants^4^) – there are signs to suggest that, on average, our participants held more favourable views. For instance, our overall sample mean score for support was 3.67 on a 5-point scale, while Kohl and colleagues’ (2020) mean score for support (for ‘gene editing invasive/non-native animals to keep them from reproducing’) was 3.88 on a 7-point scale. Furthermore, follow-up analysis of our data showed 59% supported the genetic technology (by selecting ‘4’ or ‘5’ on the 5-point scale), which is almost double the percentage (i.e., 32%) who supported gene drive for population suppression in the New Zealand-based study by MacDonald et al. (2020) and commensurate with the percentage (i.e., 61%) who supported gene drive for population suppression in non-native insect pest species (with control for spread) in the U.S.-based study by Jones et al. (2019). Arguably, the provision of the technology storyboard, which was designed to simply explain the complex technology using visual elements to support textual description, may have enhanced cognitive engagement in the decision-making exercise. Consistent with previous research, we did observe considerable variability in support and attitudes towards gene editing for the management of invasive pests – ranging from those who were more negative and non-supportive, to those who were more positive and supportive. We note though, that longitudinal research and standardised research methods, would be required to make more definitive claims about how public perceptions differ across studies performed in different countries, and over time.

Importantly, our study extends beyond simply measuring attitudes and support, by 1) segmenting people into homogeneous groups based on their support and attitudinal profile and 2) exploring whether and how these groups may differ in their engagement beliefs and preferences, and information needs. In doing so, our study aims to provide real-world, practical guidance to scientists and science directors, communicators, and policymakers alike, on the different types of communication topics, and engagement pathways that will be required to reach a broader and more diverse audience base, and thereby lead to more equalitarian input into decision-making about the use of genetic technology for invasive pest management.

A small (∼6%) yet distinctive segment, labelled Certain Objectors, held negative attitudes and feelings about the technology, and expressed the lowest support for its development. While this segment held the most pro-environmental attitudes and were highly aware of the pest problem, they did not perceive pests as more than a moderate threat. Certain Objectors were very concerned about the potential for gene editing to be improperly used and result in negative long-term impacts. They did not hold a great deal of trust in scientists to develop the technology responsibly, nor did they trust the government agencies who would be responsible for monitoring and regulating the technology. It is also noted that Certain Objectors were less undecided about the technology, which, as a reflection of attitude certainty, might suggest that their attitudes would be more persistent and resistant to persuasion (Petrocelli et al., 2007; Rucker et al., 2014; Tormala, 2016; Tormala and Petty, 2002; 2004).

There was a sizeable portion (∼26%) who held highly positive views towards the gene editing technology, and they too felt less undecided about the technology. We labelled this segment the Certain Supporters and, in all regards, their attitudes, beliefs, and perceptions towards the technology were on the positive end of the spectrum. They believed the solution to be effective, exciting, and ethical. They also held the highest trust in scientists and government agencies. Certain Supporters also were highly problem aware and perceived pests to be a significant threat. The remaining classes included *Fence Sitters* (∼31%) who could be described as expressing mainly middle-ground attitudes; and Cautious Supporters (∼38%) who expressed attitudes that were more favourable than *Fence Sitters* but not quite as favourable as Certain Supporters. Both groups felt moderately undecided about the technology, which, as a reflection of attitude uncertainty, suggests that their attitudes may be relatively easier to change (Petrocelli et al., 2007; Rucker et al., 2014; Tormala, 2016; Tormala and Petty, 2002; 2004).

### 4.1 Belief in public engagement, engagement preferences, and information needs

In terms of beliefs regarding appropriate levels of public involvement in decisions about the technology, the current study found that most Australians surveyed believed the public should be consulted with and their opinions considered (42%) (or at least kept informed, 38%), while fewer people thought the public should be directly involved (11%), or not involved at all (3%). This pattern of results almost mimics those observed in the 2021 Special Eurobarometer survey measuring EU citizens’ knowledge and attitudes towards science and technology (European Commission, 2021). In that survey, only 8% thought that public opinion should be the main concern when making decisions, while 32% indicated the public should be consulted with and their opinions seriously considered, 52% indicated the public should be informed, and 7% felt the public did not need to be involved. Our study of course, showed that these beliefs varied markedly across different segments in the population. Perhaps the most remarkable difference was between Certain Objectors and the remaining classes in terms of “direct involvement” – almost half of the Certain Objectors believed the public should be directly involved while only around 1 in ten of the other classes held the same belief. The one form of public engagement approach that many people ‘supported’ across all classes was for the consultative engagement approach–which lends support for the value of the ‘leaving ajar’ approach as explained by Russell et al. (2022).

We found around 40% placed some importance on having a say in how the technology would be developed/implemented. A similar proportion wanted to access information or provide feedback through social media, or to receive results of research. Yet the desire to participate in more intensive forms of engagement (e.g., participating in information sessions, and formally contributing to decisions through written submissions) was substantially less. The relatively lower popularity for more intensive engagement lends support to early discussions that questioned the effectiveness of participatory approaches to engaging with the public (Sturgis, 2014). Instead, what we observed was that lighter touch, less onerous public engagement approaches might be more appropriate when considering a general audience – at least in the initial stages of introducing the idea of new technologies and solutions where the goal of public engagement may be to simply raise public awareness of the problem at hand, and potential solutions. There were some differences in engagement preferences across classes, though within each class, people still prioritised the ‘light touch’ approaches of simply receiving results of research or accessing information or providing feedback through social media. Certainly, social media channels offer clear advantages over more intensive public engagements – they are an easy, cheap, and flexible vehicle through which people can learn, interact with others, and provide feedback. Our results accord with the literature, which has highlighted the usefulness of social media for engaging with the public on science matters (Hargittai et al., 2018; Oliver et al., 2023; Stilgoe et al., 2014), especially since people are increasingly using the Internet and social media channels as their primary source of information about science and technology (National Science Board, 2016). Yet it is important to recognise that there may be challenges associated with using social media channels to engage the public on science-related matters – it is an uncontrollable space after all, and one that engages users in brief, rapid, and transient ways (Huber et al., 2019). One challenge identified in the literature is how to present scientific information along with important contextual detail (Ophir and Jamieson, 2021), while other challenges relate to the presence of scientific misinformation (Vraga and Bode, 2017) and incivility that often emerges in social media discussions about science (Anderson and Huntington, 2017). Despite these challenges, a cross-country study has shown that social media news use is positively associated with trust in science (Huber et al., 2019). This finding suggests that it may well be worth tackling the challenges and investing more effort into social media-enabled public engagement exercises, including those that may involve intensive two-way dialogue (Huber et al., 2019; Oliver et al., 2023). However, it remains to be seen whether people will be interested in participating in such engagements.

One of the most remarkable findings across classes was that Certain Objectors reported a strong need to have a say, with almost 75% indicating that it was important for them to be involved this way. In contrast, the remaining classes seemed only moderately interested. The comparatively stronger interest in being involved among Certain Objectors was also observed for all engagement options listed. These results suggest that opportunities for public engagement are more likely to be taken up by a small and unique segment of the population – a group of people who greatly care for the environment but who also hold negative and potentially immovable views towards the technology and who distrust the scientists and governing bodies involved in the technology’s development and regulation. The relatively stronger interest in having a say, and being engaged, among Certain Objectors may be partly explained by the fact that as people become more certain of their attitudes, they become increasingly willing to talk about their views, and engage in advocacy, including making efforts to persuade others to adopt their views (Akhtar et al., 2013; Cheatham and Tormala, 2015). Yet, interestingly, *Certain Supporters*, who also reported more certain attitudes, did not express as strong an interest in having a say and engaging on the technology. This raises the possibility that there also needs to be a compelling reason for people to feel sufficiently motivated to translate their certainly felt attitudes into advocacy-related actions. For instance, as reflected in their attitudinal profile, Certain Supporters were the most comfortable and at ease with the technology, showing the highest level of trust in those who would be responsible for developing and managing it. Thus, it could be hypothesised that because they trusted the experts, they felt they could defer to the experts, reducing the need for their personal involvement. In contrast, Certain Objectors felt the entire opposite about scientists, government, and regulation, which may be the necessary catalyst to incense them into being involved in future engagements – hence why the marked interested in having a say across the range of engagements listed. Future research may wish to explore the underlying reasons and goals for choosing to participate or not, in engagement activities among participants to more clearly establish the motivational basis for and against participation. The question also remains as to who should facilitate these engagement activities given that Certain Objectors – who presumably might form a higher proportion of the audience given their stronger interest in having a say – do not trust the scientists or government agencies involved in the technology’s development. Rather than scientists or government agencies co-ordinating engagements, it may be more effective for representatives from environmental organisations to lead such initiatives. Certain Objectors might be more receptive to communications coming from environmental organisations (such as the Australian Wildlife Conservancy) because such organisations work to protect the environment and accordingly hold the same pro-environmental attitudes as Certain Objectors. Thus, environmental organisations may be viewed as more credible, competent, ethical, and just. Future research should examine how trust might vary across a broader range of information sources or engagement facilitators, including environmental organisations.

The results revealed most people wanted to know about the possible risks, and what was being done to regulate and control the technology; they were less interested in knowing about the claimed benefits. Also, there was some interest in knowing more about the scientific processes and techniques, who is funding the research and why, who will benefit and who will bear the risks, and what is being done to deal with the social and ethical issues involved. Interestingly, roughly 20% did not actually need or want to know anything more. The relatively stronger focus on possible risks and risk management is consistent with earlier studies in the context of gene editing for conservation purposes (Jones et al., 2019; Kohl et al., 2019; MacDonald et al., 2021a). We also identified differences across the groups in information needs. Certain Objectors were more interested in fundamental social and ethical issues, whereas Certain Supporters were more interested in knowing about scientific processes and techniques. Overall, the results suggest the need for a broader conversation about the possible risks, what is being done to regulate and control the technology, and what is being done to deal with the social and ethical issues. Information about scientific processes and techniques could be made available for an interested subgroup but is unlikely to be something of broad appeal.

When designing such information, it is important to keep in mind that how messages are framed, and the specific words and language used, can affect people in different ways. Consistent with the emotions-as-frames model (Nabi, 2003), messages that communicate the benefits (i.e., a gain frame) tend to elicit positive emotions, which in turn is associated with openness to new information and experiences; while messages that convey the negatives, downsides or risks (i.e., a loss frame) tend to trigger negative emotions and an ensuing narrowing of thought and actions (Bilandzic et al., 2020; Fredrickson and Branigan, 2005; Nabi et al., 2019). Thus, if information is to be designed to deliver on people’s information needs – that is, to know more about risks and the regulation and controls surrounding the technology – consideration should be given to the potential for raising negative emotions. Relatedly, careful thought should be given to the language or words used when talking about pest species, as certain terms can be value-laden and/or metaphorical, strongly eliciting certain attitudes and emotions. For example, the term ‘invasive’ generated more support for action (both in terms of the use of poison and gene editing to reduce reproduction) to control non-native species, as compared to the term ‘non-native’ (Kohl et al., 2020).

## 5 Conclusion and next steps

This study provides insight into the types of engagement activities and information that may be used by research scientists, technology developers and regulators when first introducing the idea of genetic technology for pest control to the public, and when seeking feedback and input from the public on whether, and how the technology should be developed and deployed. Our findings are relevant to countries elsewhere in the world that are experiencing similar invasive pest problems in terms of impacts on native flora and fauna, and agricultural industry, and that are considering innovative solutions in genetic technology.

While our results suggest most people should feel satisfied with ‘light touch’ engagement such as accessing information via social media and receiving a summary of research results, it is noted that there may still be a place for more intensive engagement activities such as in-person meetings and written submissions. However, these latter types of engagements are expected to attract a smaller but more vocal segment of the public who may already hold entrenched negative attitudes. As such, these engagements will need to be handled sensitively and with an understanding that they may not necessarily result in a democratic or representative viewpoint being established. Given the desire for greater involvement, it is possible that participatory dialogue via social media platforms, may offer another way to meet the needs of this group – however, more research is required to test and optimise these methods. In fact, more research is required to explore the potential of digital communication channels given they naturally have broader reach and thereby enable the voice of diverse audiences to be heard. More research could also be undertaken to explore other ‘light touch’ engagement approaches such as social science surveys (of the like administered in the current study), which can also capture views from a representative sample of the population for incorporation into research and science planning and decision-making (Sturgis, 2014). While ‘participating in research surveys’ were not presented as an option in our question assessing public engagement preferences, we did pose a separate question at the close of our survey, which asked people if they would be willing to be contacted for future research on new synthetic biology. Here, we found 54% of the sample were willing, and the percentage in the affirmative was consistently high across all classes (ranging from 43% of Fence Sitters to 66% of Certain Supporters). While recognising the biased nature of the sample (i.e., individuals who have already registered to take part in research surveys) and the generic nature of the question, it does provide some suggestion that surveys could be another viable ‘light touch’ option for engaging with the public on new technology.

Some limitations should be considered in interpreting the results. First, the information presented to people was quite general and broad in nature – they were presented with a hypothetical future-oriented technological solution for a problem facing the nation at large; the solution was framed in a non-specific, generic way (i.e., genes of cats/carp could be modified); there was no information provided about the potential risks of the genetic technology; and nor did we ask people to evaluate alternative pest control strategies currently used. People may hold entirely different views depending on the salience, immediacy, and specificity of the problem, the perceived need for a genetic solution, the nature and description of the solution/s presented to them, and the stated purpose of engagement. Future research may therefore wish to provide more contextual detail, perhaps through scenarios, to increase the ecological validity of the decision-making situation. Alternatively, future research may wish to conduct similar studies in situations where a specific animal pest problem is already highly personally relevant and meaningful – such as in countries and communities where certain pests (e.g., feral cats) are a known problem and where the regulatory climate is open to considering genetic solutions. These place-based assessments could be designed to evaluate a broader suite of pest management options side-by-side, including different genetic modification applications (e.g., gene editing to disrupt reproduction vs. reduce survival) and current pest control methods such as trapping, shooting and baiting. The findings from such research should more closely correspond to real-world responses. It should also be noted that our results are specific to a sample of the general population in Australia in 2018. Interest in, and views towards, the topic may be different now, especially since most people throughout the world have now been exposed to mRNA vaccines (i.e., the COVID-19 vaccines). Additionally, there have been advancements in the technology since 2018, such as the identification of alternative approaches to limit the spread of genetic changes (Johnson et al., 2024), though most laboratory research is still confined to insect pest species (Grilli et al., 2021; Legros et al., 2021; Seydel, 2024; Yan et al., 2023) with challenges continuing in mice model work (Grunwald et al., 2019; Legros et al., 2021). Considering the time lag, and intervening technological change, new social science studies are required to provide more contemporaneous understanding of public opinion.

Overall, our results shed light on how different segments in the population may respond to, and want to be engaged in, proposals to genetically modify invasive animal pest species – at least in the initial stages of introducing such an innovation. It is hoped that our results will spur further research into new, alternative, and inventive public engagement pathways – purpose-built to meet not only the needs of the scientists, technology developers and regulators, but also to meet the needs and preferences of the wider population.

## Data Availability

The datasets analysed in this article are not readily available because participants did not specifically consent to their data being shared. Requests to access the data will be considered and should be directed to the corresponding author and CSIRO's ethics committee on csshrec@csiro.au quoting the number 013/18.

## Footnotes

1 For example, research has shown more support: 1) for gene editing in invasive/non-native animal species to disrupt reproduction vs. reduce survival (Kohl et al., 2020), 2) for gene editing (to disrupt reproduction) in invasive animal species vs. non-native animal species (Kohl et al., 2020), 3) for gene drives to alter insect pests so they no longer carry a crop disease vs. reduce their population (Jones et al., 2019), 4) for gene drives in non-native (vs. native) insect pests (Jones et al., 2019), and 5) for gene drives where controls (vs. no controls) are in place to limit the spread of the drive (Jones et al., 2019).

2 For example, research has shown more support: 1) for gene editing in invasive/non-native animal species among males and individuals with greater trust in scientists and government (Kohl et al., 2020), 2) for gene drives in insect pests among individuals who do not seek out non-GMO food (Jones et al., 2019), 3) for gene editing in wildlife conservation (which included both editing genes to improve endangered species’ chances for survival and editing genes to decrease or eliminate local populations of invasive, non-native species) among those who more strongly believed in the authority of scientific knowledge, and who did not believe as strongly that gene editing is messing with nature (Kohl et al., 2019) and 4) for gene drives in animal pests among groups of individuals with a scientific worldview (MacDonald et al., 2020; 2021).

3 While the survey was conducted at the end of 2018, we have prepared earlier publications based on the survey data including the following organisational technical report: Synthetic Biology Platform (2020). Public perceptions of using synthetic biology to manage invasive pests. CSIRO, Australia; and the following journal article: Hobman, E. V., Mankad, A., & Carter, L. (2022). Public perceptions of synthetic biology solutions for environmental problems. Frontiers in Environmental Science, 10, https://doi.org/10.3389/fenvs.2022.928732.

4
MacDonald, Edwards, et al. (2021) and MacDonald et al. (2020) examined gene drive for population suppression in animals (rats, stoats and wasps) using both infertility and death examples, and in comparison to other pest control methods (the Trojan female technique and a species-specific toxin); Jones et al. (2019) examined gene drive for population suppression in insect pest species, and to alter a population of insects to not transmit crop diseases; Kohl et al. (2019) examined gene editing (with the potential for the genetic change to be passed on) to decrease or eliminate local populations of animals or plants causing environmental problems, and to improve endangered plants’ and animals’ chances for survival; and Kohl et al. (2020) examined gene editing in non-native/invasive animal species to keep from reproducing or to reduce their ability to survive’.
