# Supplementary material for: Public attitudes to genetic technology for invasive pest control and preferences for engagement and information: a segmentation analysis
Source: Front Bioeng Biotechnol. 2025 Jan 22;12:1388512. doi: 10.3389/fbioe.2024.1388512 (PMC11794500; doi:10.3389/fbioe.2024.1388512)
Supplement: Supplementary file 1 [file Table1.docx]

Supplemental Table 1 Means, standard deviations, and intercorrelations among the variables used in the segmentation analysis

|  | Mean (SD) | 1 | 2 | 3 | 4 | 5 | 6 | 7 | 8 | 9 | 10 | 11 | 12 | 13 | 14 | 15 | 16 | 17 |
| --- | --- | --- | --- | --- | --- | --- | --- | --- | --- | --- | --- | --- | --- | --- | --- | --- | --- | --- |
| 1 Pro-environmental attitude | 3.68 (0.55) |  |  |  |  |  |  |  |  |  |  |  |  |  |  |  |  |  |
| 2 Problem awareness | 3.91 (1.14) | 0.19‡ |  |  |  |  |  |  |  |  |  |  |  |  |  |  |  |  |
| 3 Threat severity | 4.10 (0.97) | 0.18‡ | 0.60‡ |  |  |  |  |  |  |  |  |  |  |  |  |  |  |  |
| 4 Comprehension | 2.55 (0.81) | 0.18‡ | 0.38‡ | 0.37‡ |  |  |  |  |  |  |  |  |  |  |  |  |  |  |
| 5 Subjective understanding | 3.23 (0.94) | 0.10† | 0.37‡ | 0.27‡ | 0.30‡ |  |  |  |  |  |  |  |  |  |  |  |  |  |
| 6 Attitude – evaluative | 3.54 (1.01) | -0.07* | 0.21‡ | 0.37‡ | 0.18‡ | 0.29‡ |  |  |  |  |  |  |  |  |  |  |  |  |
| 7 Attitude – value-based | 3.45 (1.08) | -0.08† | 0.24‡ | 0.38‡ | 0.14‡ | 0.23‡ | 0.84‡ |  |  |  |  |  |  |  |  |  |  |  |
| 8 Response efficacy | 3.90 (0.95) | 0.07* | 0.39‡ | 0.53‡ | 0.43‡ | 0.41‡ | 0.57‡ | 0.50‡ |  |  |  |  |  |  |  |  |  |  |
| 9 Relative advantage | 3.80 (0.98) | 0.06* | 0.32‡ | 0.45‡ | 0.30‡ | 0.35‡ | 0.61‡ | 0.55‡ | 0.67‡ |  |  |  |  |  |  |  |  |  |
| 10 Concern regarding dual use | 3.38 (1.02) | 0.18‡ | -0.02 | -0.13‡ | 0.04 | -0.09† | -0.44‡ | -0.43‡ | -0.24‡ | -0.28‡ |  |  |  |  |  |  |  |  |
| 11 Concern regarding long-term impacts | 3.28 (0.97) | 0.17‡ | -0.04 | -0.17‡ | -0.003 | -0.15‡ | -0.54‡ | -0.52‡ | -0.33‡ | -0.36‡ | 0.67‡ |  |  |  |  |  |  |  |
| 12 Positive affect | 3.39 (0.93) | 0.04 | 0.32‡ | 0.44‡ | 0.23‡ | 0.38‡ | 0.63‡ | 0.58‡ | 0.57‡ | 0.61‡ | -0.21‡ | -0.31‡ |  |  |  |  |  |  |
| 13 Negative affect | 2.29 (0.95) | 0.07* | -0.17‡ | -0.27‡ | -0.19‡ | -0.12‡ | -0.54‡ | -0.52‡ | -0.40‡ | -0.43‡ | -0.43‡ | 0.52‡ | -0.31‡ |  |  |  |  |  |
| 14 Support for development of gene drive technology | 3.67 (1.08) | -0.01 | 0.28‡ | 0.40‡ | 0.26‡ | 0.32‡ | 0.75‡ | 0.70‡ | 0.60‡ | 0.65‡ | -0.38‡ | -0.49‡ | 0.68‡ | -0.51‡ |  |  |  |  |
| 15 Undecided about the technology | 2.61 (1.15) | -0.01 | -0.18‡ | -0.19‡ | -0.08† | -0.31‡ | -0.32‡ | -0.32‡ | -0.25‡ | -0.24‡ | 0.32‡ | 0.39‡ | -0.21‡ | 0.29‡ | -0.30‡ |  |  |  |
| 16 Trust in scientists | 3.47 (0.99) | -0.02 | 0.25‡ | 0.25‡ | 0.22‡ | 0.31‡ | 0.58‡ | 0.53‡ | 0.52‡ | 0.52‡ | -0.37‡ | -0.40‡ | 0.53‡ | -0.41‡ | 0.58‡ | -0.21‡ |  |  |
| 17 Trust in government agency | 3.07 (1.04) | -0.10‡ | 0.13‡ | 0.22‡ | 0.09** | 0.29‡ | 0.54‡ | 0.48‡ | 0.43‡ | 0.45‡ | -0.37‡ | -0.37‡ | 0.48‡ | -0.30‡ | 0.50‡ | -0.17‡ | 0.68‡ |  |
| 18 Confidence in regulation | 3.33 (0.97) | -0.10‡ | 0.15‡ | 0.28‡ | 0.14‡ | 0.30‡ | 0.58‡ | 0.54‡ | 0.51‡ | 0.55‡ | -0.40‡ | -0.44‡ | 0.53‡ | -0.35‡ | 0.57‡ | -0.22‡ | 0.64‡ | 0.67‡ |

*Note.*  * p<0.001. † p<0.01. ‡ p<0.001. Except for comprehension, all variables were measured using a 5-point Likert-scale. Comprehension scores ranged from 0 to 3.
