# Supplementary material for: Public attitudes to genetic technology for invasive pest control and preferences for engagement and information: a segmentation analysis
Source: Front Bioeng Biotechnol. 2025 Jan 22;12:1388512. doi: 10.3389/fbioe.2024.1388512 (PMC11794500; doi:10.3389/fbioe.2024.1388512)
Supplement: Supplementary file 2 [file Table2.docx]

Supplemental Table 2 Latent profile fit statistics and expected proportion in the population and expected sample number (based on posterior probability)

| Number of profiles | Number of free parameters (df) | Log-likelihood | AIC | BIC | EXPECTED POPULATION PROPORTION  (EXPECTED SAMPLE N) | | | | | |  |
| --- | --- | --- | --- | --- | --- | --- | --- | --- | --- | --- | --- |
|  |  |  |  |  | Profile 1 | Profile 2 | Profile 3 | Profile 4 | Profile 5 | Profile 6 | unallocated |
| 1 | 36 | -28535.98 | 57143.96 | 57325.64 | 100%  n=1,149 |  |  |  |  |  | 0.0%  n=0 |
| 2 | 55 | -25805.64 | 51721.27 | 51998.84  ↓9.29% | 45.00%  n=517 | 55.00%  n=632 |  |  |  |  | 0.0%  n=0 |
| 3 | 74 | -24854.69 | 49857.38 | 50230.83  ↓3.40% | 8.09%  n=93 | 47.17%  n=542 | 44.73%  n=514 |  |  |  | 0.0%  n=0 |
| 4 | 93 | -24275.72 | 48737.44 | 49206.77  ↓2.04% | 5.83%  n=67 | 30.90%  n=355 | 37.60%  n=432 | 25.67%  n=295 |  |  | 0.0%  n=0 |
| 5 | 112 | -23772.93 | 47769.86 | 48335.09  ↓1.77% | 5.57%  n=64 | 11.05%  n=127 | 24.02%  n=276 | 36.55%  n=420 | 22.63%  n=260 |  | 0.17%  n=2 |
| 6 | 131 | -23572.62 | 47407.25 | 48068.36  ↓0.55% | 5.48%  n=63 | 7.66%  n=88 | 24.11%  n=277 | 6.01%  n=69 | 33.68%  n=387 | 22.80%  n=262 | 0.26%  n=3 |
