# Supplementary material for: Public attitudes to genetic technology for invasive pest control and preferences for engagement and information: a segmentation analysis
Source: Front Bioeng Biotechnol. 2025 Jan 22;12:1388512. doi: 10.3389/fbioe.2024.1388512 (PMC11794500; doi:10.3389/fbioe.2024.1388512)
Supplement: Supplementary file 3 [file Table5.docx]

Supplementary Material

Identification and description of classes (see Table 1 and Table 2)

*Certain Objectors* (n=67, 5.83%), which we labelled *Certain Objectors*, comprised the smallest percentage of respondents. These participants rated themselves as significantly more pro-environmental (Mean=3.90) than *Fence Sitters* (Mean=3.64, Bonferroni t=-3.48, p=0.003, Cohen’s d=0.50), *Cautious Supporters* (Mean=3.67, Bonferroni t=-3.12, p=0.011, Cohen’s d=0.43) and *Certain Supporters* (Mean=3.69, Bonferroni t=-2.83, p=0.028, Cohen’s d=0.34). They also reported greater awareness of the invasive pest problem (Mean=3.87) compared to *Fence Sitters* (Mean=3.29, Bonferroni t=-4.15, p<0.001, Cohen’s d=0.47), less awareness compared to *Certain Supporters* (Mean=4.51, Bonferroni t=4.56, p<0.001, Cohen’s d=0.74) and similar awareness to *Cautious Supporters* (Mean=4.01). *Certain Objectors* participants also perceived invasive pests were less of a problem (Mean=3.42), compared to *Cautious Supporters* (4.28, Bonferroni t=7.91, p<0.001, Cohen’s d=1.07) and *Certain Supporters* participants (Mean=4.72, Bonferroni t=11.52, p<0.001, Cohen’s d=1.84). In terms of comprehension scores, *Certain Objectors* participants achieved a lower score (Mean=2.40) than *Certain Supporters* participants (Mean=2.86, Bonferroni t=4.47, p<0.001, Cohen’s d=0.95) but were no different to *Fence Sitters* or *Cautious Supporters* participants. *Certain Objectors* participants also rated themselves as less knowledgeable of the synthetic biology solution (Mean=3.37) compared to *Certain Supporters* participants (Mean=3.89, Bonferroni t=4.68, p<0.001, Cohen’s d=0.58). However, *Certain Objectors* participants thought they were more knowledgeable than *Fence Sitters* participants (Mean=2.69, Bonferroni t=6.22, p<0.001, Cohen’s d=0.81). They also held the most negative evaluative attitudes towards the solution (Mean=1.40), scoring significantly less than *Fence Sitters* (Mean=2.84, Bonferroni t=19.71, p<0.001, Cohen’s d=2.47), *Cautious Supporters* (Mean=3.73, Bonferroni t=32.35, p<0.001, Cohen’s d= 3.98) and *Certain Supporters* participants (Mean=4.58, Bonferroni t=42.76, p<0.001, Cohen’s d=7.17). Similarly, they also reported the most negative value-based attitudes (Mean=1.37), significantly less than *Fence Sitters* (Mean=2.71, Bonferroni t=15.37, p<0.001, Cohen’s d=2.04), *Cautious Supporters* (Mean=3.63, Bonferroni t=26.14, p<0.001, Cohen’s d=3.31), and *Certain Supporters* participants (Mean=4.54, Bonferroni t=35.59, p<0.001, Cohen’s d=5.42). When it came to rating whether the solution would be effective in reducing or eliminating invasive pests, *Certain Objectors* participants considered it would not be as effective (Mean=2.55), as compared to *Fence Sitters* (Mean=3.23, Bonferroni t=7.51, p<0.001, Cohen’s d=0.81), *Cautious Supporters* (Mean=4.05, Bonferroni t=17.00, p<0.001, Cohen’s d=2.16), and *Certain Supporters* (Mean=4.79, Bonferroni t=24.55, p<0.001, Cohen’s d=3.87). Similarly, *Certain Objectors* participants rated its relative advantage over current methods lower (Mean=2.04) than *Fence Sitters* (Mean=3.19, Bonferroni t=12.93, p<0.001, Cohen’s d=1.44), *Cautious Supporters* (Mean=3.95, Bonferroni t=21.75, p<0.001, Cohen’s d=2.65), and *Certain Supporters* (Mean=4.72, Bonferroni t=29.55, p<0.001, Cohen’s d=4.04). In terms of concerns regarding dual use, *Certain Objectors* participants held greater concern (Mean=4.61) compared to *Fence Sitters* (Mean=3.69, Bonferroni t=-7.72, p<0.001), *Cautious Supporters* (Mean=3.40, Bonferroni t=-10.41, p<0.001) and *Certain Supporters* (Mean=2.68, Bonferroni t=-16.06, p<0.001). They also were more concerned about the long-term impacts (Mean=4.69) compared to *Fence Sitters* (Mean=3.65, Bonferroni t=-9.95, p<0.001, Cohen’s d=1.26), *Cautious Supporters* (Mean=3.32, Bonferroni t=-13.38, p<0.001, Cohen’s d=1.96) and *Certain Supporters* (Mean=2.47, Bonferroni t=-21.02, p<0.001, Cohen’s d=2.79). In terms of their feelings regarding the solution, *Certain Objectors* participants reported less positive affect (Mean=1.91) than *Fence Sitters* (Mean=2.75, Bonferroni t=9.54, p<0.001, Cohen’s d=1.18), *Cautious Supporters* (Mean=3.61, Bonferroni t=19.58, p<0.001, Cohen’s d=2.71), and *Certain Supporters* (Mean=4.16, Bonferroni t=25.02, p<0.001, Cohen’s d=3.37). Conversely, they reported more negative affect (Mean=3.85) compared to *Fence Sitters* (Mean2.72, Bonferroni t=-11.11, p<0.001, Cohen’s d=1.40), *Cautious Supporters* (Mean=2.17, Bonferroni t=16.80, p<0.001, Cohen’s d=2.16) and *Certain Supporters* (Mean=1.61, Bonferroni t=-21.75, p<0.001, Cohen’s d=2.88). They reported the lowest level of support (Mean=1.40), which was significantly different to *Fence Sitters* (Mean=2.87, Bonferroni t=18.87, p<0.001, Cohen’s d=2.35), *Cautious Supporters* (Mean=3.96, Bonferroni t=33.33, p<0.001, Cohen’s d=4.18), and *Certain Supporters* (Mean=4.74, Bonferroni t=42.22, p<0.001, Cohen’s d=6.71). They also reported feeling less undecided (Mean=1.97) than *Fence Sitters* (Mean=3.38, Bonferroni t=12.45, p<0.001, Cohen’s d=1.50) and *Cautious Supporters* (Mean=2.75, Bonferroni t=7.69, p<0.001, Cohen’s d=0.96), but more undecided when compared to *Certain Supporters* (Mean=1.51, Bonferroni t=-2.76, p=0.035, Cohen’s d=0.40). *Certain Objectors* participants were less trusting of scientists (Mean=1.70) than *Fence Sitters* (Mean=2.94, Bonferroni t=12.76, p<0.001, Cohen’s d=1.59), *Cautious Supporters* (Mean=3.63, Bonferroni t=20.20, p<0.001, Cohen’s d=2.71), and *Certain Supporters* (Mean=4.28, Bonferroni t=26.22, p<0.001, Cohen’s d=3.48). A similar pattern also was observed for trust in government whereby *Certain Objectors* reported less trust (Mean=1.40) compared to *Fence Sitters* (Mean=2.61, Bonferroni t=10.89, p<0.001, Cohen’s d=1.49), *Cautious Supporters* (Mean=3.19, Bonferroni t=16.42, p<0.001, Cohen’s d=2.21), and *Certain Supporters* (Mean=3.84, Bonferroni t=21.73, p<0.001, Cohen’s d=2.93). Relatedly, *Certain Objectors* participants also reported less confidence in regulation surrounding the solution (Mean=1.66), compared to *Fence Sitters* (Mean=2.82, Bonferroni t=12.17, p<0.001, Cohen’s d=1.57), *Cautious Supporters* (Mean=3.42, Bonferroni t=18.70, p<0.001, Cohen’s d=2.46), and *Certain Supporters* (Mean=4.19, Bonferroni t=26.04, p<0.001, Cohen’s d=3.42).

*Fence Sitters* (n=355, 30.90%), which we labelled *Fence Sitters*, comprised approximately one-third of respondents. *Fence Sitters* held moderate-to-high pro-environmental attitudes (Mean=3.64), like that observed in *Cautious Supporters* and *Certain Supporters*, but significantly less than *Certain Objectors* (Mean=3.90, Bonferroni t=-3.48, p=0.003, Cohen’s d=0.50). *Fence Sitters* participants were less aware of the pest problem (Mean=3.29), when compared to *Certain Objectors* (Mean=3.87, Bonferroni t=-4.15, p<0.001, Cohen’s d=0.47), *Cautious Supporters* (Mean=4.01, Bonferroni t=9.71, p<0.001, Cohen’s d=0.65), and *Certain Supporters* (Mean=4.51, Bonferroni t=14.86, p<0.001, Cohen’s d=1.15). *Fence Sitters* participants rated the problem of pests (Mean=3.50) much like *Certain Objectors* (Mean=3.42, Bonferroni t=0.78, ns) participants, which was significantly less than *Cautious Supporters* (Mean=4.01, Bonferroni t=13.06, p<0.001, Cohen’s d=0.88) and *Certain Supporters* (Mean=4.51, Bonferroni t=18.47, p<0.001, Cohen’s d=1.42). *Fence Sitters* participants achieved a similar comprehension score (Mean=2.19) to *Certain Objectors* (Mean=2.40, Bonferroni t=-2.11, ns), which was significantly lower than *Cautious Supporters* (Mean=2.66, Bonferroni t=8.62, p<0.001, Cohen’s d=0.55) and *Certain Supporters* (Mean=2.86, Bonferroni t=11.25, p<0.001, Cohen’s d=0.83). *Fence Sitters* participants reported less subjective understanding (Mean=2.69) compared to *Certain Objectors* (Mean=3.37, Bonferroni t=-6.22, p<0.001, Cohen’s d=0.81), *Cautious Supporters* (Mean=3.19, Bonferroni t=8.38, p<0.001, Cohen’s d=0.63) and *Certain Supporters* (Mean=3.89, Bonferroni t=18.55, p<0.001, Cohen’s d=1.44). Their evaluative attitudes were more positive (Mean=2.84) than *Certain Objectors* (Mean=1.40, Bonferroni t=19.71, p<0.001, Cohen’s d=2.47), but less positive than *Cautious Supporters* (Mean=3.73, Bonferroni t=22.65, p<0.001, Cohen’s d=1.51) and *Certain Supporters* (Mean=4.58, Bonferroni t=40.12, p<0.001, Cohen’s d=3.35). A similar pattern was observed for value-based attitudes whereby *Fence Sitters* participants reported more favourable attitudes (Mean=2.71) compared to *Certain Objectors* (Mean=1.37, Bonferroni t=15.37, p<0.001, Cohen’s d=2.04), but less favourable attitudes compared to *Cautious Supporters* (Mean=3.63, Bonferroni t=19.34, p<0.001, Cohen’s d=1.32) and *Certain Supporters* (Mean=4.54, Bonferroni t=35.14, p<0.001, Cohen’s d=2.85). *Fence Sitters* participants reported greater belief that the solution would be effective (Mean=3.23) compared to *Certain Objectors* (Mean=2.55, Bonferroni t=7.51, p<0.001, Cohen’s d=0.81) but less belief compared to *Cautious Supporters* (Mean=4.05, Bonferroni t=17.18, p<0.001, Cohen’s d=1.16) and *Certain Supporters* (Mean=4.79, Bonferroni t=29.46, p<0.001, Cohen’s d=2.39). Similarly, *Fence Sitters* participants thought it would be more advantageous compared to other solutions (Mean=3.19), than did *Certain Objectors* (Mean=2.04, Bonferroni t=12.93, p<0.001, Cohen’s d=1.44), but less advantageous compared to *Cautious Supporters* (Mean=3.95, Bonferroni t=15.83, p<0.001, Cohen’s d=1.13) and *Certain Supporters* (Mean=4.72, Bonferroni t=15.15, p<0.001, Cohen’s d=2.43). *Fence Sitters* participants were less concerned about dual use (Mean=3.69) than were *Certain Objectors* (Mean=4.61, Bonferroni t=-7.72, p<0.001, Cohen’s d=1.01), but were more concerned than *Cautious Supporters* (Mean=3.40, Bonferroni t=-4.72, p<0.001, Cohen’s d=0.34) and *Certain Supporters* (Mean=2.68, Bonferroni t=-10.67, p<0.001, Cohen’s d=1.10). The same pattern was observed for concern regarding long-term impacts – *Fence Sitters* participants showed less concern (Mean=3.65) than *Certain Objectors* (Mean=4.69, Bonferroni t=-9.95, p<0.001, Cohen’s d=1.26), but greater concern than *Cautious Supporters* (Mean=3.32, Bonferroni t=-6.02, p<0.001, Cohen’s d=0.44) and *Certain Supporters* (Mean=2.47, Bonferroni t==19.28, p<0.001, Cohen’s d=1.42). *Fence Sitters* participants felt more positive (Mean=2.75) towards to the solution, compared to *Certain Objectors* (Mean=1.91, Bonferroni t=9.54, p<0.001, Cohen’s d=1.18) but less positive compared to *Cautious Supporters* (Mean=3.61, Bonferroni t=18.14, p<0.001, Cohen’s d=1.30) and *Certain Supporters* (Mean=4.16, Bonferroni t=26.85, p<0.001, Cohen’s d=2.04). The same pattern was observed for negative feelings, with *Fence Sitters* participants reporting less negative affect (Mean=2.72) compared to *Certain Objectors* (Mean=3.85, Bonferroni t=-11.11, p<0.001, Cohen’s d=1.40) but more negative affect compared to *Cautious Supporters* (Mean=2.17, Bonferroni t=-10.13, Cohen’s d=0.73) and *Certain Supporters* (Mean=1.61, Bonferroni t=-18.59, p<0.001, Cohen’s d=1.49). *Fence Sitters* participants were more supportive of the development of the solution (Mean=2.87) compared to *Certain Objectors* (Mean=1.40, Bonferroni t=18.87, p<0.001, Cohen’s d=2.35), but were less supportive than either *Cautious Supporters* (Mean=3.96, Bonferroni t=26.01, p<0.001, Cohen’s d=1.76) or *Certain Supporters* (Mean=4.74, Bonferroni t=40.62, p<0.001, Cohen’s d=3.32). *Fence Sitters* participants were the most undecided about the solution (Mean=3.38), reporting greater undecidedness compared to *Certain Objectors* (Mean=1.97, Bonferroni t=12.45, p<0.001, Cohen’s d=1.50), *Cautious Supporters* (Mean=2.75, Bonferroni t=-9.05, p<0.001, Cohen’s d=0.63) and *Certain Supporters* (Mean=1.51, Bonferroni t=-25.79, p<0.001, Cohen’s d=2.12). Trust in scientists was greater (Mean=2.94) than *Certain Objectors* (Mean=1.70, Bonferroni t=12.76, p<0.001, Cohen’s d=1.59) but less than *Cautious Supporters* (Mean=3.63, Bonferroni t=13.31, p<0.001, Cohen’s d=0.96) and *Certain Supporters* (Mean=4.28, Bonferroni t=23.47, p<0.001, Cohen’s d=1.82). The same pattern also was observed for trust in the government agency – *Fence Sitters* showed higher trust (Mean=2.61) than *Certain Objectors* (Mean=1.40, Bonferroni t=10.89, p<0.001, Cohen’s d=1.49) but less trust than *Cautious Supporters* (Mean=3.19, Bonferroni t=9.84, p<0.001, Cohen’s d=0.71) and *Certain Supporters* (Mean=3.84, Bonferroni t=18.91, p<0.001, Cohen’s d=1.46). *Fence Sitters* participants also expressed greater confidence in regulation (Mean=2.82) compared to *Certain Objectors* (Mean=1.66, Bonferroni t=12.17, p<0.001, Cohen’s d=1.57) but less confidence than *Cautious Supporters* (Mean=3.42, Bonferroni t=11.64, p<0.001, Cohen’s d=0.85) and *Certain Supporters* (Mean=4.19, Bonferroni t=24.15, p<0.001, Cohen’s d=1.90).

*Cautious Supporters* (n=432, 37.60%), which we labelled *Cautious Supporters*, comprised the largest percentage of respondents. They held lower pro-environmental attitudes (Mean=3.67) compared to *Certain Objectors* (Mean=3.90, Bonferroni t=-3.12, p=0.011, Cohen’s d=0.43), but which were similar to *Fence Sitters* (Mean=3.64) and *Certain Supporters* (Mean=3.69). They also were more aware of the pest problem (Mean=4.01) than *Fence Sitters* (Mean=3.29, Bonferroni t=9.71, p<0.001, Cohen’s d=0.65), but were less aware than *Certain Supporters* (Mean=4.51, Bonferroni t=6.29, p<0.001, Cohen’s d=0.54). *Cautious Supporters* participants rated invasive pests as more problematic (Mean=4.28) than *Certain Objectors* (Mean=3.42, Bonferroni t=7.91, p<0.001, Cohen’s d=1.07) and *Fence Sitters* (Mean=3.50, Bonferroni t=13.06, p<0.001, Cohen’s d=0.88), but less problematic than *Certain Supporters* (Mean=4.72, Bonferroni t=6.89, p<0.001, Cohen’s d=0.62). *Cautious Supporters* participants achieved similar comprehension scores (Mean=2.66) to *Certain Objectors* (Mean=2.40, Bonferroni t=-2.11, ns), greater than *Fence Sitters* (Mean=2.19, Bonferroni t=8.62, p<0.001, Cohen’s d=0.55) but less than *Certain Supporters* (Mean=2.86, Bonferroni t=3.55, p=0.002, Cohen’s d=0.35). Subjective understanding also mirrored this result whereby *Cautious Supporters* participants reported similar understanding (Mean=3.19) to *Certain Objectors* (Mean=3.37, Bonferroni t=-1.74, ns), greater understanding than *Fence Sitters* (Mean=2.69, Bonferroni t=8.38, p<0.001, Cohen’s d=0.63) and less understanding than *Certain Supporters* (Mean=3.89, Bonferroni t=11.40, p<0.001, Cohen’s d=0.87). In terms of their attitudes, they reported more favourable evaluative attitudes (Mean=3.73) compared to *Certain Objectors* (Mean=1.40, Bonferroni t=32.35, p<0.001, Cohen’s d=3.98) and *Fence Sitters* (Mean=2.84, Bonferroni t=22.65, p<0.001, Cohen’s d=1.51), but less favourable evaluative attitudes compared to *Certain Supporters* (Mean=4.58, Bonferroni t=20.37, p<0.001, Cohen’s d=1.60). A similar pattern surfaced for value-based attitudes. Here *Cautious Supporters* participants reported more favourable value-based attitudes (Mean=3.63) than *Certain Objectors* (Mean=1.37, Bonferroni t=26.14, p<0.001, Cohen’s d=3.31) and *Fence Sitters* (Mean=2.71, Bonferroni t=19.34, p<0.001, Cohen’s d=1.32), but less favourable value-based attitudes compared to *Certain Supporters* (Mean=4.54, Bonferroni t=18.32, p<0.001, Cohen’s d=1.39). In terms of whether the solution would be effective, *Cautious Supporters* participants held more favourable views (Mean=4.05) compared to *Certain Objectors* (Mean=2.55, Bonferroni t=17.00, p<0.001, Cohen’s d=2.16) and *Fence Sitters* (Mean=3.23, Bonferroni t=17.18, p<0.001, Cohen’s d=1.16), but less favourable views compared to *Certain Supporters* (Mean=4.79, Bonferroni t=14.43, p<0.001, Cohen’s d=1.31). The same pattern emerged for assessments of the solution’s relative advantage – here *Cautious Supporters* participants reported more favourable scores (Mean=3.95) than *Certain Objectors* (Mean=2.04, Bonferroni t=21.75, p<0.001, Cohen’s d=2.65) and *Cautious Supporters* (Mean=3.19, Bonferroni t=15.83, p<0.001, Cohen’s d=1.13), but less favourable scores than *Certain Supporters* (Mean=4.72, Bonferroni t=15.15, p<0.001, Cohen’s d=1.32). *Cautious Supporters* participants reported less concern regarding dual use (Mean=3.40) when compared to *Certain Objectors* (Mean=4.61, Bonferroni t=-10.41, p<0.001, Cohen’s d=1.45) and *Fence Sitters* (Mean=3.69, Bonferroni t=-4.72, p<0.001, Cohen’s d=0.34), but greater concern than *Certain Supporters* (Mean=2.68, Bonferroni t=-10.67, p<0.001, Cohen’s d=0.81). Concerns about long-term impacts also followed the same pattern with *Cautious Supporters* participants reporting less concern (Mean=3.32) than *Certain Objectors*(Mean=4.69, Bonferroni t=-13.38, p<0.001, Cohen’s d=1.96) and *Fence Sitters* (Mean=3.65, Bonferroni t=-6.02, p<0.001, Cohen’s d=0.44), but greater concern than *Certain Supporters* (Mean=2.47, Bonferroni t=-14.41, p<0.001, Cohen’s d=1.21). *Cautious Supporters* participants experienced greater positive affect (Mean=3.61) than *Certain Objectors* (Mean=1.91, Bonferroni t=19.58, p<0.001, Cohen’s d=2.71) and *Fence Sitters* (Mean=2.75, Bonferroni t=18.14, p<0.001, Cohen’s d=1.30), but less positive affect than *Certain Supporters* (Mean=4.16, Bonferroni t=10.80, p<0.001, Cohen’s d=0.85). Similarly, *Cautious Supporters* participants reported less negative affect (Mean=2.17) than *Certain Objectors* (Mean=3.85, Bonferroni t=-16.80, p<0.001, Cohen’s d=2.16) and *Fence Sitters* (Mean=2.72, Bonferroni t=-10.13, p<0.001, Cohen’s d=0.73), but more negative affect than *Certain Supporters* (Mean=1.61, Bonferroni t=-9.78, p<0.001, Cohen’s d=0.77). *Cautious Supporters* participants were more supportive of the solution (Mean=3.96) than *Certain Objectors* (Mean=1.91, Bonferroni t=33.33, p<0.001, Cohen’s d=4.18), and *Fence Sitters* (Mean=2.87, Bonferroni t=26.01, p<0.001, Cohen’s d=1.76), but less supportive than *Certain Supporters* (Mean=4.74, Bonferroni t=17.70, p<0.001, Cohen’s d=1.40). *Cautious Supporters* participants were more undecided about the solution (Mean=2.75) compared to *Certain Objectors* (Mean=1.97, Bonferroni t=7.69, p<0.001, Cohen’s d=0.96) and *Certain Supporters* (Mean=1.51, Bonferroni t=-18.32, p<0.001, Cohen’s d=1.48), but less undecided than *Fence Sitters* (Mean=3.38, Bonferroni t=-9.05, p<0.001, Cohen’s d=0.63). *Cautious Supporters* participants were more trusting of scientists (Mean=3.63) than *Certain Objectors* (Mean=1.70, Bonferroni t=20.20, p<0.001, Cohen’s d=2.71) and *Fence Sitters* (Mean=2.94, Bonferroni t=13.31, p<0.001, Cohen’s d=0.96), but less trusting than *Certain Supporters* (Mean=4.28, Bonferroni t=11.86, p<0.001, Cohen’s d=0.93). The same pattern surfaced for trust in government agency whereby *Cautious Supporters* showed greater trust (Mean=3.19) than *Certain Objectors* (Mean=1.40, Bonferroni t=16.42, p<0.001, Cohen’s d=2.21) and *Fence Sitters* (Mean=2.61, Bonferroni t=9.84, p<0.001, Cohen’s d=0.71), but less trust than *Certain Supporters* (Mean=3.84, Bonferroni t=10.40, p<0.001, Cohen’s d=0.77). Confidence in regulation also was higher for *Cautious Supporters* (Mean=3.42) compared to *Certain Objectors* (Mean=1.66, Bonferroni t=18.70, p<0.001, Cohen’s d=2.46) and *Fence Sitters* (Mean=2.82, Bonferroni t=11.64, p<0.001, Cohen’s d=0.85), but less than *Certain Supporters* (Mean=4.19, Bonferroni t=14.15, p<0.001, Cohen’s d=1.09).

*Certain Supporters* (n=295, 25.67%), which we labelled *Certain Supporters*, comprised approximately one-quarter of respondents. *Certain Supporters* participants held positive pro-environmental attitudes (Mean=3.69) equivalent to *Fence Sitters* (Mean=3.64) and *Cautious Supporters* (Mean=3.67), but significantly less than *Certain Objectors* (Mean=3.90, Bonferroni t=-3.12, p=0.028, Cohen’s d=0.34). *Certain Supporters* participants reported higher awareness of the pest problem compared to *Certain Objectors* (Mean=3.87, Bonferroni t=4.56, p<0.001, Cohen’s d=0.74), *Fence Sitters* (Mean=3.29, Bonferroni t=14.86, p<0.001, Cohen’s d=1.15), and *Cautious Supporters* (Mean=4.01, Bonferroni t=6.29, p<0.001, Cohen’s d=0.54). They considered invasive pests as a bigger problem (Mean=4.72) than *Certain Objectors* (Mean=3.42, Bonferroni t=11.52, p<0.001, Cohen’s d=1.84), *Fence Sitters* (Mean=3.50, Bonferroni t=18.47, p<0.001, Cohen’s d=1.42) and *Cautious Supporters* (Mean=4.01, Bonferroni t=6.89, p<0.001, Cohen’s d=0.62). In terms of comprehension, *Certain Supporters* participants achieved the highest score (Mean=2.86) which was significantly higher than *Certain Objectors* (Mean=2.40, Bonferroni t=4.47, p<0.001, Cohen’s d=0.95), *Fence Sitters* (Mean=2.19, Bonferroni t=11.25, p<0.001, Cohen’s d=0.83) and *Cautious Supporters* (Mean=2.66, Bonferroni t=3.55, p=0.002, Cohen’s d=0.35). Subjective understanding also followed the same pattern with *Certain Supporters* participants reporting greater understanding (Mean=3.89) than *Certain Objectors* (Mean=3.37, Bonferroni t=4.68, p<0.001, Cohen’s d=0.58), *Fence Sitters* (Mean=2.69, Bonferroni t=18.55, p<0.001, Cohen’s d=1.44) and *Cautious Supporters* (Mean=3.19, Bonferroni t=11.40, p<0.001, Cohen’s d=0.87). *Certain Supporters* participants held the most favourable evaluative attitudes towards the solution (Mean=4.58), which was significantly greater than *Certain Objectors* (Mean=1.40, Bonferroni t=42.76, p<0.001, Cohen’s d=7.16), *Fence Sitters* (Mean=2.84, Bonferroni t=40.12, p<0.001, Cohen’s d=3.35) and *Cautious Supporters* (Mean=3.73, Bonferroni t=20.37, p<0.001, Cohen’s d=1.60). Similarly, they held more favourable value-based attitudes towards the solution (Mean=4.54) as compared to *Certain Objectors* (Mean=1.37, Bonferroni t=35.59, p<0.001, Cohen’s d=5.42), *Fence Sitters* (Mean=2.71, Bonferroni t=35.14, p<0.001, Cohen’s d=2.85) and *Cautious Supporters* (Mean=3.63, Bonferroni t=18.32, p<0.001, Cohen’s d=1.39). *Certain Supporters* participants also believed that the solution would be more effective (Mean=4.79) than did *Certain Objectors* (Mean=2.55, Bonferroni t=24.55, p<0.001, Cohen’s d=3.87), *Fence Sitters* (Mean=3.23, Bonferroni t=29.46, p<0.001, Cohen’s d=2.39) and *Cautious Supporters* (Mean=4.05, Bonferroni t=14.43, p<0.001, Cohen’s d=1.31). Similarly, *Certain Supporters* participants also rated the solution as relatively more advantageous than current solutions (Mean=4.72), than did *Certain Objectors* (Mean=2.04, Bonferroni t=29.55, p<0.001, Cohen’s d=4.04), *Fence Sitters* (Mean=3.19, Bonferroni t=28.91, p<0.001, Cohen’s d=2.43) and *Cautious Supporters* (Mean=3.95, Bonferroni t=15.15, p<0.001, Cohen’s d=1.32). *Certain Supporters* participants showed some concern regarding dual use however, they were less concerned (Mean=2.68) than were *Certain Objectors* (Mean=4.61, Bonferroni t=-16.06, p<0.001, Cohen’s d=2.15), *Fence Sitters* (Mean=3.69, Bonferroni t=-14.52, p<0.001, Cohen’s d=1.10) and *Cautious Supporters* (Mean=3.40, Bonferroni t=-10.67, p<0.001, Cohen’s d=0.81). The same pattern emerged for concern regarding long-term impacts with *Certain Supporters* participants showing less concern (Mean=2.47) than *Certain Objectors* (Mean=4.69, Bonferroni t=-21.02, p<0.001, Cohen’s d=2.79), *Fence Sitters* (Mean=3.65, Bonferroni t=-19.28, p<0.001, Cohen’s d=1.42) and *Cautious Supporters* (Mean=3.32, Bonferroni t=-14.41, p<0.001, Cohen’s d=1.12). *Certain Supporters* participants felt more positive emotions (Mean=4.16) towards the solution when compared to *Certain Objectors* (Mean=1.91, Bonferroni t=25.02, p<0.001, Cohen’s d=3.37), *Fence Sitters* (Mean=2.75, Bonferroni t=26.85, p<0.001, Cohen’s d=2.04) and *Cautious Supporters* (Mean=3.61, Bonferroni t=10.80, p<0.001, Cohen’s d=0.85). Similarly, *Certain Supporters* participants reported less negative affect (Mean=1.61) than *Certain Objectors* (Mean=3.85, Bonferroni t=-21.75, p<0.001, Cohen’s d=2.88), *Fence Sitters* (Mean=2.72, Bonferroni t=-18.59, p<0.001, Cohen’s d=1.49) and *Cautious Supporters* (Mean=2.17, Bonferroni t=-9.78, p<0.001, Cohen’s d=0.77). *Certain Supporters* participants were the most supportive of the solution (Mean=4.74), showing significantly more support than *Certain Objectors* (Mean=1.40, Bonferroni t=42.22, p<0.001, Cohen’s d=6.71), *Fence Sitters* (Mean=2.87, Bonferroni t=40.62, p<0.001, Cohen’s d=3.32) and *Cautious Supporters* (Mean=3.96, Bonferroni t=17.70, p<0.001, Cohen’s d=1.40). They were the least undecided about the solution (Mean=1.51), reporting less ‘undecidedness’ than *Certain Objectors* (Mean=1.97, Bonferroni t=-2.76, p=0.035, Cohen’s d=0.40), *Fence Sitters* (Mean=3.38, Bonferroni t=-25.79, p<0.001, Cohen’s d=2.12) and *Cautious Supporters* (Mean=2.75, Bonferroni t=-18.32, p<0.001, Cohen’s d=1.48). *Certain Supporters* participants held the highest trust in scientists (Mean=4.28), which was significantly greater than *Certain Objectors* (Mean=1.70, Bonferroni t=26.22, p<0.001, Cohen’s d=3.48), *Fence Sitters* (Mean=2.94, Bonferroni t=23.47, p<0.001, Cohen’s d=1.82) and *Cautious Supporters* (Mean=3.63, Bonferroni t=11.86, p<0.001, Cohen’s d=0.93). Similarly, *Certain Supporters* participants trusted the government (Mean=3.84) more than *Certain Objectors* (Mean=1.40, Bonferroni t=21.73, p<0.001, Cohen’s d=2.93), *Fence Sitters* (Mean=2.61, Bonferroni t=18.91, p<0.001, Cohen’s d=1.46) and *Cautious Supporters* (Mean=3.19, Bonferroni t=10.40, p<0.001, Cohen’s d=0.77). *Certain Supporters* participants also felt more confident in the regulation surrounding the solution (Mean=4.19) than did *Certain Objectors* (Mean=1.66, Bonferroni t=26.04, p<0.001, Cohen’s d=3.24), *Fence Sitters* (Mean=2.82, Bonferroni t=24.15, p<0.001, Cohen’s d=1.90) and *Cautious Supporters* (Mean=3.42, Bonferroni t=14.15, p<0.001, Cohen’s d=1.09).

Class differences in demographics (see Supplemental Table 3)

We also explored differences in sex, age, education, household income, and employment across the classes. In terms of sex, results revealed that *Certain Objectors* comprised the lowest proportion of males (31.3%) and the highest proportion of females (68.7%). *Fence Sitters* also comprised proportionally fewer males (38.9%) than females (60.6%). *Certain Supporters* comprised the highest proportion of males (56.3%) and the lowest proportion of females (43.7%) and *Fence Sitters* was roughly equivalent in its proportions (45.4% male and 54.6% female). A 2 x 2 chi-square test was significant overall (Pearson χ^2^(3)=25.07, p=0.000) but the only observed frequency significantly different to that expected was observed for *Certain Supporters*. The frequency of males was significantly higher than expected (Pearson residual=2.77), and the frequency of females was significantly lower than expected (Pearson residual=-2.52).

Furthermore, group comparisons revealed that the odds of being a male 2.8 times as high in *Certain Supporters* (odds ratio=2.82, p<0.001; 56.3% were male), and 1.8 times as high in *Cautious Supporters* (odds ratio=1.82, p=0.033; 45.4% were male), when compared to *Certain Objectors* (31.3% of the group were male). The odds of being a male was also twice as high in *Certain Supporters* compared to *Fence Sitters* (odds ratio=2.02, p<0.001), and 1.5 times as high in *Certain Supporters* when compared to *Cautious Supporters* (odds ratio=1.55, p=0.004). There were no statistically significant differences in the odds of being a male between Classes 1 and 2 (31.3% versus 38.9%, respectively); and between Classes 2 and 3 (38.9% versus 45.4%, respectively).

In terms of age, a 2 x 2 chi-square test was significant overall (Pearson χ^2^(15)=101.26, p<0.001). The frequency of 18-to-24-year-old individuals was more than expected in *Fence Sitters* (Pearson residual=2.64), yet less than expected in *Certain Supporters* (Pearson residual=-2.03). Similarly, the frequency of 25-to-34-year-old individuals was more than expected in *Fence Sitters* (Pearson residual=3.25), and less than expected in *Certain Supporters* (Pearson residual=-3.21). At the other end of the age range, the frequency of 55-to-64-year-old individuals and 65 or over individuals was less than expected in *Fence Sitters* (Pearson residual=-2.51 and Pearson residual=-4.68, respectively), yet greater than expected in *Certain Supporters* (Pearson residual=3.10 and Pearson residual=3.69, respectively).

Furthermore, group comparisons revealed that *Fence Sitters* comprised a higher proportion of 18- to 24-year-old participants (17.2%) – the odds of being an 18- to 24-year-old was 2.3 times as high in *Fence Sitters* as compared to *Certain Supporters* (odds ratio=2.34, p=0.001; 8.1%), and 1.6 times as high in *Fence Sitters* as compared to *Cautious Supporters* (odds ratio=1.66, p=0.015; 11.1%). The odds of being 25-to-34-year-old was 3.4 (odds ratio=3.39, p=0.001; 23.9%), 3.2 (odds ratio=3.19, p<0.001; 22.8%) and 1.8 (odds ratio=1.77, p=0.022; 14.1%) times as high in *Certain Objectors*, *Fence Sitters* and *Cautious Supporters*, respectively, when compared to *Certain Supporters* (8.5%). Also, the odds of being 25-to-34-year-old in *Certain Objectors* and *Fence Sitters* was 1.9 (odds ratio=1.91, p=0.042; 23.9%) and 1.8 (odds ratio=1.79, p=0.002, 22.8%) times as high, respectively, when compared to *Cautious Supporters* (14.1%). The chances of being 35-to-44-year-old was twice as high in *Certain Objectors* (odds ratio=1.99, p=0.034; 25.4%), and 1.6 (odds ratio=1.57, p=0.032; 21.1%) times as high in *Fence Sitters*, when compared to *Certain Supporters* (14.6%). There were no class differences in the 45-54 age category; between 16% to 20% of respondents in each class fell into this age category. The odds of being 55 to 64 was 2.6 times as high in *Certain Supporters* (odds ratio=2.59, p=0.035; 20.3%), when compared to *Certain Objectors* (9.0%). Also, the odds of being 55 to 64 was 1.6 times as high in *Certain Supporters* (odds ratio=1.58, p=0.022; 20.3%) compared to *Cautious Supporters* (13.9%). And the odds of being 55 to 64 was 1.7 times as high in *Cautious Supporters* (odds ratio=1.69, p=0.026; 13.9%) compared to *Fence Sitters* (8.7%). The odds of being 65 or older was twice to three times as high in *Cautious Supporters* (odds ratio=2.28, p=0.028; 26.2%) and 4 (odds ratio=3.06, p=0.003; 32.2%), respectively, when compared to *Certain Objectors* (13.4%). Overall, Classes 3 and 4 tended to comprise a higher proportion of older aged participants while Classes 1 and 2 featured a higher proportion of younger aged participants.

In terms of education, a 2 x 2 chi-square test was significant overall (Pearson χ^2^(18)=38.70, p=0.003); however, there were only two significant differences between the observed and expected frequencies. The frequency of certificate holders was higher than expected in *Certain Supporters* (Pearson residual=2.52), and the frequency of bachelor degree holders was significantly less than expected in *Certain Supporters* (Pearson residual=-1.83). In terms of group differences, there was no difference among the Classes in the odds of having a Year 10 or Year 12 school education, a Diploma, or a Graduate Diploma/Certificate. However, respondents in *Certain Supporters* were twice as likely to hold a Certificate compared to respondents in *Cautious Supporters* (odds ratio=2.07, p=0.001) (19% versus 10.2%). Also, the odds of holding a bachelor’s degree was 1.5 times as great for respondents in *Fence Sitters* (odds ratio=1.49, p=0.034; 26.8%) and *Cautious Supporters* (odds ratio=1.52, p=0.022; 27.1%) compared to *Certain Supporters* (19.7%). The odds of holding a postgraduate degree was 1.6 times higher for respondents in *Cautious Supporters* (odds ratio=1.58, p=0.028; 17.1%) compared to *Fence Sitters* (11.6%), and 1.8 times higher for respondents in *Cautious Supporters* (odds ratio=1.83, p=0.009) compared to *Certain Supporters* (10.2%). Overall, these results suggest that participants in *Cautious Supporters* might be slightly more well educated than the other Classes, though the relatively small sample size for *Certain Objectors* reduces our confidence in the comparisons involving *Certain Objectors*.

For household income, the observed frequencies were not significantly different to that expected across income categories and classes (Pearson χ^2^(21)=28.89, p=0.117). Only a few significant group differences were observed. The odds of earning between $150,000 to $199,999/year was twice as great for respondents in *Certain Supporters* (odds ratio=2.04, p=0.016; 10.9%) compared to those in *Fence Sitters* (5.6%). The odds of earning $300,000 or more/yr was 2.5 times as great for respondents in *Cautious Supporters* (odds ratio=2.53, p=0.052; 4.2%) compared to those in *Fence Sitters* (1.7%). The odds of preferring not to say was 1.9 times as great for respondents in *Fence Sitters* (odds ratio=1.89, p=0.004; 19.4%) compared to those in *Certain Supporters* (11.9%).

For employment, a 2 x 2 chi-square test was significant overall (Pearson χ^2^(9)=30.34, p<0.001). However, only two significant differences were observed – the frequency of those unemployed was significantly higher than that expected in *Fence Sitters* (Pearson residual=2.08), yet the frequency of those not in the labour force was significantly less than expected in *Fence Sitters* (Pearson residual =-2.40). In terms of group differences, the odds of being employed was 1.6 times greater for respondents in *Fence Sitters* (odds ratio=1.63, p=0.003; 66.2%) and 1.4 times greater for respondents in *Cautious Supporters* (odds ratio=1.36, p=0.045; 62.0%), when compared to *Certain Supporters* (54.6%). The odds of being unemployed was 2.5 times as great for respondents in *Fence Sitters* (odds ratio=2.54, p=0.013; 8.2%) compared to *Certain Supporters* (3.4%). The odds of not being in the labour force was 2.5 times greater for respondents in *Certain Supporters* (odds ratio=2.46, p=0.011; 32.5%) compared to *Certain Objectors* (16.4%), 2.3 times greater for respondents in *Certain Supporters* (odds ratio=2.33, p<0.001) compared to *Fence Sitters* (17.2%), and 1.6 times greater for respondents in *Certain Supporters* (odds ratio=1.6, p=0.005) compared to *Cautious Supporters* (23.2%).

Class differences in beliefs and preferences concerning public engagement activities

Table 3 presents the mean scores and the percentage of respondents who scored above the mid-point on the scale (the percentages provide an indication of the level of belief in the appropriate level of public involvement in decision making about the technology, and interest in participating in different types of engagement activities, and how this interest varies across the classes). Inspection of the means indicates that overall, participants generally felt that moderate levels of public involvement were appropriate (Mean=2.65); they themselves were moderately interested in receiving results of research (Mean=3.17) and accessing information and/or providing feedback through social media (Mean=3.10). There was slightly less interest in formally contributing to decisions (Mean=2.48) and participating in public information sessions (Mean=2.68). In this regard, significance testing revealed that participants were more interested in receiving results of research than accessing information and providing feedback through social media, though this effect was small (t_(1148)_=2.07, p=0.039, Cohen’s d=0.06). They also were more interested in receiving results of research than participating in public information sessions, with this effect being moderate (t_(1148)_=14.40, p=0.000, Cohen’s d=0.42). Participants also indicated greater interest in receiving results of research than formally contributing to decision-making, with this effect being moderate (t_(1148)_=21.43, p=0.000, Cohen’s d=0.63). Similarly, participants were more interested in accessing information and providing feedback through social media than in participating in public information sessions (t_(1148)_=11.94, p=0.000, Cohen’s d=0.35) or formally contributing to decision-making (t_(1148)_=17.75, p=0.000, Cohen’s d=0.52).

In terms of class differences in the means, statistically significant effects were observed for the engagement beliefs and preferences questions. First, we observed that *Certain Objectors* (Mean=3.38) believed the public should be more heavily involved in decisions compared to all other classes (*Fence Sitters* Mean=2.85, Bonferroni t=-5.63, p<0.001, Cohen’s d=0.78; *Cautious Supporters* Mean=2.59, Bonferroni t=-8.55, p<0.001, Cohen’s d=1.17; *Certain Supporters* Mean=2.37, Bonferroni t=-10.60, p<0.001, Cohen’s d=1.41). In fact, close to half of *Certain Objectors* indicated that the public should be directly involved in decision-making, whereas only a small percentage (6.7% - 12.7%) of respondents in the other classes reported this view. Many *Certain Objectors* also favoured the public being consulted and considered (35.8%). *Fence Sitters* and *Cautious Supporters* tended to believe the public either should be consulted with, and their views considered (48.5% and 46.5%, respectively) or simply kept informed (23.4% and 41.2%, respectively). *Certain Supporters* also tended to favour public being kept informed (55.6%) and consulted and considered (29.8%). Belief in public involvement in decision-making also was statistically significantly different between all the remaining classes, with *Fence Sitters* reporting greater public involvement than *Cautious Supporters* (Bonferroni t=5.00, p<0.001) and *Certain Supporters* (Bonferroni t=-8.46, p<0.001); and *Cautious Supporters* reporting greater public involvement than *Certain Supporters* (Bonferroni t=-4.16, p<0.001).

Similarly, we observed that the importance placed on being personally involved in decision-making was highest in *Certain Objectors* (Mean=4.04) compared to all other classes (*Fence Sitters* Mean=3.23, Bonferroni t=-5.71, p<0.001, Cohen’s d=0.84; *Cautious Supporters* Mean=3.08, Bonferroni t=-6.90, p<0.001, Cohen’s d=0.95; *Certain Supporters* Mean=2.77, Bonferroni t=-8.78, p<0.001, Cohen’s d=1.04). In fact, the majority (74.6%) of *Certain Objectors* respondents scored above the mid-point of the scale, whereas less than half of respondents in the other classes scored above the mid-point. Respondents in *Fence Sitters* and *Cautious Supporters* also placed a significantly higher level of importance on being involved in decision-making when compared to respondents in *Certain Supporters* (Bonferroni t=-5.42, p<0.001, Cohen’s d=0.41; Bonferroni t=-3.74, p=0.001, Cohen’s d=0.27, respectively).

A similar pattern of results was observed for all the types of decision-making approaches listed. For *participating in public information sessions*, participants in *Certain Objectors* reported a greater desire to do so (Mean=3.37), when compared to those in *Fence Sitters* (Mean=2.78, Bonferroni t=-3.73, p=0.001, Cohen’s d=0.53), *Cautious Supporters* (Mean=2.65, Bonferroni t=-4.62, p<0.001, Cohen’s d=0.60), and *Certain Supporters* (Mean=2.45, Bonferroni t=-5.66, p<0.001, Cohen’s d=0.69). In fact, close to half (47.8%) of participants in *Certain Objectors* scored above the mid-point on the scale, whereas only around one-fifth to a quarter of participants in the remaining classes scored above the mid-point on the scale. Respondents in *Fence Sitters* also reported a greater desire to participate in public information sessions compared to those in *Certain Supporters*, though this effect was small (Bonferroni t=-3.42, p=0.004, Cohen’s d=0.27).

For *accessing information/providing feedback through social media*, respondents in *Certain Objectors* reported a greater desire to do so (Mean=3.97) when compared to *Fence Sitters* (Mean=3.04, Bonferroni t=-5.74, p<0.001, Cohen’s d=0.80), *Cautious Supporters* (Mean=3.11, Bonferroni t=-5.35, p<0.001, Cohen’s d=0.73), and *Certain Supporters* (Mean=2.96, Bonferroni t=-6.10, p<0.001, Cohen’s d=0.76). Here, around 70% of participants in *Certain Objectors* scored above the mid-point on the scale, whereas less than half of respondents in the other Classes scored above the mid-point on the scale.

Regarding *formally contributing to decisions*, respondents in *Certain Objectors* reported a greater desire to do so (Mean=3.28) when compared to those in *Fence Sitters* (Mean=2.68, Bonferroni t=-3.74, p=0.001, Cohen’s d=0.51), *Cautious Supporters* (Mean=2.42, Bonferroni t=-5.47, p<0.001, Cohen’s d=0.71) and *Certain Supporters* (Mean=2.16, Bonferroni t=-6.88, p<0.001, Cohen’s d=0.84). Around half (50.8%) of respondents in *Certain Objectors* scored above the mid-point on the scale, whereas around one-fifth of respondents in the other classes scored above the mid-point on the scale. Further differences, albeit smaller, were observed between *Fence Sitters* and 3 (Bonferroni t=-3.06, p=0.013, Cohen’s d=0.23), *Fence Sitters* and 4 (Bonferroni t=-5.49, p<0.001, Cohen’s d=0.44), and *Cautious Supporters* and 4 (Bonferroni t=-2.83, p=0.029, Cohen’s d=0.21).

Regarding *receiving results of the research (e.g., summary report)*, respondents in *Certain Objectors* again reported a greater desire for this to occur (Mean=3.85) compared to *Fence Sitters* (Mean=3.06, Bonferroni t=-4.89, p<0.001, Cohen’s d=0.66), *Cautious Supporters* (Mean=3.15, Bonferroni t=-4.39, p<0.001, Cohen’s d=0.59), and *Certain Supporters* (Mean=3.20, Bonferroni t=-3.92, p=0.001, Cohen’s d=0.49). Around two-thirds (67.2%) of participants in *Certain Objectors* scored higher than the mid-point of the scale, whereas less than half of participants in the remaining classes scored higher than the mid-point.

Overall, these results suggest that participants in *Certain Objectors* place high importance on, and indicate most interest in, participating in decision-making. By comparison, the remaining participants reported moderate importance, and moderate-to-less interest in participating in decision-making.

Class differences in information needs

Figure 1 reports the type of information wanted by participants. It presents the percentage of respondents within each class who selected a given information option as a ‘top 3 topic/issue’ they would like to hear more about.

Across the entire sample, information about the possible risks of the proposed technology was rated by the greatest number of participants (n=695, 61%). Information about what is being done to regulate and control the technology also was rated by many participants (n=595, 52%) as being in their ‘top 3’ information needs. On the other end of the spectrum, a smaller number (n=235, 20%) indicated that they wanted to know what the claimed benefits are. And a small proportion (n=210, 18%) also did not need or want to know anything more about the technology. This pattern of results suggest that people may be more focussed on risk and risk management, than they are on understanding the benefits – although it is possible that most people did not want to know about the benefits because the storyboard had provided a summary of the key benefits already.

We explored group differences in information needs. There were some notable differences across classes in the types of information nominated.

For *scientific processes and techniques*, respondents in *Certain Supporters* had greater odds of selecting this option (43.4% selected it) compared to all other classes. Respondents in *Certain Supporters* were 3.9 times as likely to select it compared to *Certain Objectors* (odds ratio=3.90, p<0.001; 16.4%), 3.1 times as likely to select it compared to *Fence Sitters* (odds ratio=3.12, p<0.001; 19.7%) and twice as likely to select it compared to *Cautious Supporters* (odds ratio=2.02, p<0.001; 28.1%). Respondents in *Cautious Supporters* also had greater odds of selecting this option compared to *Fence Sitters* (odds ratio=1.5, p=0.011). Interestingly, regarding *who is funding the research and why?* there were no class differences observed. Across classes, there was approximately 25% who indicated this was a top 3 information need. Regarding *claimed benefits*, participants in *Fence Sitters* (odds ratio=2.99, p=0.024; 19.4%), *Cautious Supporters* (odds ratio=3.59, p=0.008; 22.5%), and *Certain Supporters* (odds ratio=3.44, p=0.011; 21.7%) all had greater odds of wanting to know what the claimed benefits are, as compared to participants in *Certain Objectors* (7.5% selected it). Similar results were observed for ‘possible risks’ wherein participants in *Fence Sitters* (odds ratio=1.81, p=0.027; 59.4%), *Cautious Supporters* (odds ratio=1.90, p=0.015; 60.7%), and *Certain Supporters* (odds ratio=2.30, p=0.002; 65.1%) all had greater odds of wanting to know what the possible risks are, as compared to participants in *Certain Objectors* (44.8% selected it).

For *who will benefit and who will bear the risks*, participants in *Cautious Supporters* (33.6%) had greater odds of selecting this option compared to those in *Certain Supporters* (odds ratio=1.54, p=0.011; 24.8%). For *regulation and control of technology*, participants in *Cautious Supporters* (56.0%) and *Certain Supporters* (55.6%) had greater odds of selecting this option when compared to *Fence Sitters* (odds ratio=1.59, p=0.001, and odds ratio=1.56, p=0.005, respectively; 44.5%).

Regarding *social and ethical issues*, participants in *Certain Objectors* had greater odds of selecting this option (49.3% selected it), when compared to *Fence Sitters* (odds ratio=2.13, p=0.005; 31.3%), *Cautious Supporters* (odds ratio=2.23, p=0.003; 30.3%) and *Certain Supporters* (odds ratio=3.80, p<0.001; 20.3%). Additionally, participants in *Fence Sitters* (odds ratio=1.78, p=0.002) and those in *Cautious Supporters* (odds ratio=1.70, p=0.003) had greater odds of selecting this option when compared to *Certain Supporters*. And finally, for *not needing or wanting more information*, participants in *Certain Objectors* had greater odds of selecting this option (26.9% selected it) when compared to *Cautious Supporters* (odds ratio=2.19, p=0.011; 14.4%) and *Certain Supporters* (odds ratio=2.28, p=0.011; 13.9%). Participants in *Fence Sitters* also had greater odds of selecting this option (25.1% selected it) when compared to *Cautious Supporters* (odds ratio=2.00, p<0.001; 14.4%) and *Certain Supporters* (odds ratio=2.07, p<0.001; 13.9%).
