# Supplementary material for: Public attitudes to genetic technology for invasive pest control and preferences for engagement and information: a segmentation analysis
Source: Front Bioeng Biotechnol. 2025 Jan 22;12:1388512. doi: 10.3389/fbioe.2024.1388512 (PMC11794500; doi:10.3389/fbioe.2024.1388512)
Supplement: Supplementary file 4 [file Table3.docx]

Supplemental Table 3 Demographics across the 4 classes

|  | OVERALL SAMPLE  *n*=1149 | CLASS 1  *Certain dissenters*  *n*=67 (5.83%) | CLASS 2  *Cautious moderates*  *n*=355 (30.90%) | CLASS 3  *Cautious supporters n*=432 (37.60%) | CLASS 4  *Certain supporters*  *n*=295 (25.67%) |
| --- | --- | --- | --- | --- | --- |
| Sex |  |  |  |  |  |
| Male | 521 (45.3%) | 21 (31.3%) | 138 (38.9%) | 196 (45.4%) | 166 (56.3%) |
| Female | 626 (54.5%) | 46 (68.7%) | 215 (60.6%) | 236 (54.6%) | 129 (43.7%) |
| Other | 2 (0.2%) |  | 2 (0.6%) |  |  |
| Age group |  |  |  |  |  |
| 18-24 years | 141 (12.3%) | 8 (11.9%) | 61 (17.2%) | 48 (11.1%) | 24 (8.1%) |
| 25-34 years | 183 (15.9%) | 16 (23.9%) | 81 (22.8%) | 61 (14.1%) | 25 (8.5%) |
| 35-44 years | 216 (18.8%) | 17 (25.4%) | 75 (21.1%) | 81 (18.8%) | 43 (14.6%) |
| 45-54 years | 198 (17.2%) | 11 (16.4%) | 70 (19.7%) | 69 (16.0%) | 48 (16.3%) |
| 55-64 years | 157 (13.7%) | 6 (9.0%) | 31 (8.7%) | 60 (13.9%) | 60 (20.3%) |
| 65 or over years | 254 (22.1%) | 9 (13.4%) | 37 (10.4%) | 113 (26.2%) | 95 (32.2%) |
| Education |  |  |  |  |  |
| School education (Year 10 or below) | 113 (9.8%) | 4 (6.0%) | 45 (12.7%) | 37 (8.6%) | 27 (9.2%) |
| School education | 171 (14.9%) | 11 (16.4%) | 57 (16.1%) | 58 (13.4%) | 45 (15.3%) |
| Certificate | 156 (13.6%) | 8 (11.9%) | 48 (13.5%) | 44 (10.2%) | 56 (19.0%) |
| Advanced Diploma/ Diploma | 191 (16.6%) | 14 (20.9%) | 51 (14.4%) | 67 (15.5%) | 59 (20.0%) |
| Bachelor degree | 287 (25.0%) | 17 (25.4%) | 95 (26.8%) | 117 (27.1%) | 58 (19.7%) |
| Graduate Diploma/ Graduate certificate | 74 (6.4%) | 1 (1.5%) | 18 (5.1%) | 35 (8.1%) | 20 (6.8%) |
| Postgraduate degree | 157 (13.7%) | 12 (17.9%) | 41 (11.6%) | 74 (17.1%) | 30 (10.2%) |
| Household income |  |  |  |  |  |
| less than $50,000/yr | 271 (23.6%) | 15 (22.4%) | 88 (24.8%) | 99 (22.9%) | 69 (23.4%) |
| $50,000 - $99,999/yr | 308 (26.8%) | 19 (28.4%) | 88 (24.8%) | 125 (28.9%) | 76 (25.8%) |
| $100,000 - $149,999/yr | 188 (16.4%) | 7 (10.5%) | 67 (18.9%) | 60 (13.9%) | 54 (18.3%) |
| $150,000 - $199,999/yr | 92 (8.0%) | 8 (11.9%) | 20 (5.6%) | 32 (7.4%) | 32 (10.9%) |
| $200,000 - $249,999/yr | 45 (3.9%) | 3 (4.5%) | 10 (2.8%) | 19 (4.4%) | 13 (4.4%) |
| $250,000 - $299,999/yr | 21 (1.8%) | 1 (1.5%) | 4 (1.1%) | 9 (2.1%) | 7 (2.4%) |
| $300,000 or more/yr | 34 (3.0%) | 1 (1.5%) | 6 (1.7%) | 18 (4.2%) | 9 (3.1%) |
| Prefer not to say | 190 (16.5%) | 13 (19.4%) | 72 (20.3%) | 70 (16.2%) | 35 (11.9%) |
| Employment |  |  |  |  |  |
| Employed (full-time or part-time) | 707 (61.5%) | 43 (64.2%) | 235 (66.2%) | 268 (62.0%) | 161 (54.6%) |
| Unemployed, looking for work | 64 (5.6%) | 4 (6.0%) | 29 (8.2%) | 21 (4.9%) | 10 (3.4%) |
| Not in labour force | 268 (23.3%) | 11 (16.4%) | 61 (17.2%) | 100 (23.2%) | 96 (32.5%) |
| Other | 110 (9.6%) | 9 (13.4%) | 30 (8.5%) | 43 (10.0%) | 28 (9.5%) |
