# Supplementary material for: Public attitudes to genetic technology for invasive pest control and preferences for engagement and information: a segmentation analysis
Source: Front Bioeng Biotechnol. 2025 Jan 22;12:1388512. doi: 10.3389/fbioe.2024.1388512 (PMC11794500; doi:10.3389/fbioe.2024.1388512)
Supplement: Supplementary file 5 [file Table4.docx]

Method

Participants

A demographically representative sample of 1,149 Australians participated in this study. Imposed quotas ensured that the sample was representative of the national population on age (18-24 years: 12.3%; 25-34 years: 15.9%; 35-44 years: 18.8%, 45-54 years: 17.2%, 55-64 years: 13.7%, 65 years or over, 22.1%) and gender (male: 45.3%, female: 54.5%, other: 0.2%). A range of education (*Mode:* Bachelor degree: 25.0%), and income levels (*Mode:* $50,000 to $99,999 per annum: 26.8%) were represented in the sample, and approximately 67.1% of the sample was either employed or looking for work.

Procedure

Participants were recruited via an external third-party research agency, with each participant receiving a token incentive for survey completion. To participate in the study, respondents were required to be an Australian resident over the age of 18 years. The study was conducted during a 3-week period from November to December, 2018. The project received ethical clearance from the CSIRO Social and Interdisciplinary Science Human Research Ethics Committee.

A standard introductory email was sent to potential participants, inviting them to take part in an online survey. Once participants clicked on the link to the survey, an information page was displayed, which explained the purpose of the study (to better understand what the public thinks about new biotechnology) and invited individuals to participate in the survey. Those that agreed to participate indicated consent by ticking a checkbox and continuing with the survey. Demographic information (age, gender, postcode, and state of residence) was collected at the commencement of the survey to monitor and achieve demographic quotas, thereby ensuring a representative sample of the Australian population on age, gender, and location. Towards the start of the survey, participants were provided with a general definition of synthetic biology:

- Synthetic biology is a new field of research bringing together genetics, chemistry and engineering. It allows scientists to design and build new biological organisms, so that they may perform new functions.
- Synthetic biology can use DNA to create new characteristics, or remove certain functions, in plants, animals and other organisms (e.g., bacteria, fungi, algae).

Additionally, a pop-up box also provided the following definition of DNA for those who hovered over the word ‘DNA’:

- DNA are molecules that carry genetic instructions used in development, general functioning and reproduction in all living things.

Participants then received information on the problem of invasive pest species in Australia and a possible synthetic biology solution (i.e., gene editing of invasive pests). A power-point style presentation, or ‘technology storyboard’, was presented to participants to convey this information. The storyboard provided both textual and visual information about the novel technological solution. Two sex-biasing examples were provided: (1) the genes of feral cats could be modified so that all offspring are a single sex (e.g., male only), reducing opportunities to mate and decreasing the population over time, and (2) the genes of European carp could be modified so that females only produce infertile males, reducing carp numbers over several generations. The storyboard was developed by the authors in collaboration with biotechnical scientists who were engaged in the early-stage science underpinning the technology, as well as CSIRO communication specialists (to view the technology storyboard, see <https://research.csiro.au/synthetic-biology-fsp/public-attitudes/>).

Survey

The survey included questions designed to measure a range of variables deemed to be of interest to the study of public perceptions of a novel synthetic biology solution for invasive pest management. In terms of overall structure, the survey first presented information about synthetic biology and a specific synthetic biology solution as applied to the problem of invasive pests, and asked respondents several questions about their views towards this solution. Demographic information was collected both at the beginning (to address quota requirements) and end of the survey. All questions included a 5-point Likert-style response scale, unless otherwise noted. The survey took on average 15 minutes to complete.

We were highly selective in the choice of indicators for inclusion in our segmentation analysis. It was important that the subgroups would not only be well distinguished by the indicators, but that such group distinctions would be meaningful. As such, we selected indicators that have a strong conceptual basis in terms of prior research demonstrating their importance in explaining public attitudes and responses to new technology. Also, since prior research has shown that using more indicators in latent class analysis is generally beneficial (e.g., it decreases the occurrence of solutions with low class assignment accuracy, improves convergence rates, and reduces parameter bias) (Wurpts & Geiser, 2014), we used several indicators to assist in the identification of reliable latent classes or subgroups. Extending on previous public perception research in synthetic biology and invasive pests (MacDonald et al., 2020), we included the following variables to serve as indicators in a latent profile analysis.

**Pro-environmental attitude**

*Pro-environmental attitude* or an ecological worldview was measured using 9 items from the 15-item revised New Ecological Paradigm (Dunlap et al., 2000). These items were selected based on their applicability to the context of humans introducing novel technologies into the natural environment. The items assessed included: (1) anti-anthropocentrism – the rejection of the belief that nature exists primarily for human use (e.g., ‘Plants and animals have as much right as humans to exist’); (2) fragility of nature’s balance – the belief that the balance of nature is threated by human activities (e.g., ‘When humans interfere with nature it often produces disastrous consequences’); and (3) anti-exemptionalism – the rejection of the belief that humans, unlike other species, are exempt from the constraints of nature (e.g., ‘Despite our special abilities humans are still subject to the laws of nature’). Participants indicated their agreement with these statements. The items were coded so that high scores reflected a pro-environmental attitude. When the three facets were kept separate, the internal consistency reliability was not adequate (Cronbach’s alpha=0.63 for Anti-anthropocentrism; Cronbach’s alpha=0.58 for Balance of Nature; and Cronbach’s alpha=0.43 for Anti-exemptionalism). A Principal Components Analysis (PCA) revealed that all items loaded on a single factor, and the internal consistency reliability was acceptable (Cronbach’s alpha = 0.76) (Cortina, 1993). On average, participants held a pro-environmental attitude (Mean=3.68, SD=0.55).

**Perceptions of the pest problem**

*Problem awareness* was measured by asking participants ‘Before today, to what extent were you aware that invasive pests are present in Australia?’ (1=no awareness, 3=medium awareness, 5=high awareness). On average, participants were quite aware of the problem of invasive pests (Mean=3.91, SD=1.14). *Threat severity* was measured by asking participants ‘To what extent do you think invasive pests are a problem?’ (1=not a problem at all to 5=a very big problem). On average, participants rated invasive pests as a big problem (Mean=4.10, SD=0.97). These questions were developed from Protection Motivation Theory, which explains how fear appeals and coping processes influence attitude change (Rogers R, 1975, 1983).

**Understanding of the gene editing solution**

*Comprehension* of the information contained in the animation was measured by asking participants three true or false questions, two of which were true: ‘Invasive pests include wild rabbits, feral cats and wild dogs, among others’, ‘Gene editing could involve modifying genes so that animals only produce male offspring’ and one which was false: ‘Gene editing of invasive pest species aims to increase the population of invasive pests)’. Correct answers were summed with scores ranging from 0 to 3. The average comprehension score was 2.55 (SD=0.81).

*Subjective understanding* was measured with the following question ‘Based on the information provided and your own general knowledge, how well do you feel you understand what this technology is about?’ (1=not very well, 3=moderately well, 5=very well). On average, participants felt that they moderately understood what the technology is about (Mean=3.23, SD=0.94).

**Attitudes**

Drawing on literature examining the nature of attitudes (Ajzen, 1991, 2008), participants were asked to provide an overall favourable or unfavourable evaluation of the genetic technology.

*Evaluative attitudes* – or one’s overall favourable or unfavourable evaluation of the gene drive solution through a consideration of its outcomes – was assessed with 3 semantic differential statements. Participants were asked ‘Thinking about your overall impressions of this technology, use the scales below to provide a rating for the technology based on each set of adjectives. I feel this technology would be: 1=harmful……5=beneficial; 1=bad…..5=good; and 1=risky…..5=safe’. Scores were combined to provide an assessment of global attitude towards the gene drive solution. The internal consistency reliability was acceptable (Cronbach’s alpha = 0.88). On average, participants rated the technology just above the mid-point of the scale (Mean=3.54, SD=1.01).

*Value-based attitudes* – or one’s overall favourable or unfavourable evaluation of the gene drive solution through the consideration of ethics and morality – was assessed with 2 semantic differential statements. Participants were asked ‘Thinking about your overall impressions of this technology, use the scales below to provide a rating for the technology based on each set of adjectives. I feel this technology would be: 1=unethical…..5=ethical; and 1=immoral….5=moral’. On average, participants rated the technology just above the mid-point of the scale (Mean=3.45, SD=1.08).

*Attitude undecidedness or ambivalence.* We also measured the extent to which participants felt undecided about the technology ‘To what extent do you feel undecided about this technology?’ (1=not at all to 5=very much) (Priester & Petty, 1996). On average, participants tended to feel less undecided (Mean=2.61, SD=1.15).

**Perceived effectiveness**

*Response efficacy* – or one’s belief that the solution will be effective (an important component of the protection motivation theory (Rogers, 1975, 1983)) – was assessed by asking participants ‘To what extent do you believe this new technology would help reduce or eliminate invasive pests?’ (1=would not help at all to 5=would be very helpful). On average, participants felt that the technology would result in this outcome (Mean=3.90, SD=0.95).

*Relative advantage* (a key characteristic within the diffusion of innovation theory: (Rogers, 2003) was measured by asking participants ‘I think that this new technology would be better than current methods of invasive pest management’ (1=strongly disagree to 5=strongly agree). On average, participants slightly agreed that they technology would be better than current methods of invasive pest management (Mean=3.80, SD=0.98).

**Concerns regarding improper use and long-term impacts**

Concerns regarding improper use and long-term impacts of genetic technology were measured by presenting participants with a pre-specified list of general concerns. In the absence of established questions, we developed these questions for the purposes of the study.

*Concern regarding improper use* was measured with two statements ‘To what extent would you be concerned that the technology could get into the wrong hands and be used for bad purposes’ and ‘To what extent would you be concerned that the technology could be inadvertently misused, leading to unintended negative consequences’ (1=not concerned to 5=extremely concerned). These items were highly correlated (*r*=0.79) and combined to provide a measure of concern regarding improper use. On average, participants showed moderate concern regarding the technology’s potential improper use (Mean=3.38, SD=1.02).

*Concern regarding long-term impacts* was measured with three statements about the impacts on humans/animals (‘To what extent would you be concerned about the long-term effects of the technology on humans and animals?’), the environment (‘To what extent would you be concerned about the long-term effects of the technology on the natural environment?’), and whether the consequences can be effectively controlled (‘To what extent would you be concerned about whether the consequences of the technology can be effectively controlled or managed?’) (1=not concerned, 3=moderately concerned, 5=extremely concerned). The three items were combined to provide an overall assessment of concern regarding the long-term impacts. The internal consistency reliability was acceptable (Cronbach’s alpha = 0.89) (Cortina, 1993). On average, participants were moderately concerned about the impacts (Mean=3.28, SD=0.97).

**Emotions**

*Positive and negative affect* were measured by asking participants ‘When you read through the information about this technology, to what extent did it make you feel: hopeful, excited, curious, concerned, afraid, and angry’ (1=not at all to 5=very much) (adapted from the Positive and Negative Affect Schedule: Watson et al. (1988)). The ratings for hopeful, excited, and curious were combined to provide a measure of positive affect (Cronbach’s alpha=0.80), and the ratings for concerned, afraid, and angry were combined to measure negative affect (Cronbach’s alpha=0.80). On average, participants experienced moderate positive affect (Mean=3.39, SD=0.93) and less negative affect (Mean=2.29, SD=0.95).

**Support**

*Support for development of the gene drive technology* was measured by asking participants ‘Overall, based on the information provided and your own general knowledge, to what extent would you support the development of this technology?’ (1=would not support to 5=would strongly support). On average, participants were more supportive than unsupportive (Mean=3.67, SD=1.08).

**Trust and confidence in governance**

Drawing on research examining the role of social trust in those who manage hazards (Siegrist & Cvetkovich, 2000), t*rust in scientists* was measured by asking ‘How much do you trust that scientists working on this technology would develop it responsibly?’ (1=no trust, 3=moderate trust, 5=high trust). On average, participants showed moderate trust in scientists (Mean=3.47, SD=0.99). *Trust in government* was measured by asking ‘How much do you trust the government agency that would be responsible for approving and regulating this technology – for example, the Office of the Gene Technology Regulator?’ (1=no trust, 3=moderate trust, 5=high trust). On average, participants moderately trusted the government agency (Mean=3.07, SD=1.04).

*Confidence in regulation* was measured by asking participants to rate their level of agreement (1=strongly disagree to 5=strongly agree) with two statements: ‘I think that this technology will be well regulated’, and ‘I think legislation and regulation can be counted on to ensure that this technology is developed in a safe way’ (adapted from Zhang et al. (2018)). These two measures were combined to assess overall confidence in regulation (Cronbach’s alpha=0.87). On average, participants very slightly agreed with these statements (Mean=3.33, SD=0.97).

**Public engagement beliefs and preferences, and information needs**

In addition to the indicator variables that were used to identify segments based on attitudes towards the technology and its development, we also measured how important participants felt it was to be personally involved in decision-making regarding the technology, their engagement preferences, and what further information they would like to know. These variables were kept separate from the main segmentation analysis so that we could explore in greater depth differences between the groups on participation, engagement, and information needs.

*Belief in appropriate public involvement in decision making* was measured with the question ‘Which of the following most accurately reflects your feelings about the appropriate level of public involvement when it comes to making decisions about this technology?’ (adapted from a Eurobarometer survey: European Commission 2012, 2021). The responses provided included:

- ‘the public does not need to be involved in decisions about this technology’
- ‘the public should be kept informed of decisions made about this technology’
- ‘the public should be consulted with, and their opinions considered, when making decisions about this technology’
- ‘the public should be directly involved in making decisions about this technology’

A ‘don’t know’ option was also provided.

*Importance of having a say* was measured with the questions ‘How important would it be for you to have a say in how this technology is developed?’ and ‘How important would it be for you to have a say in how this technology is implemented?’ (1=not important to 5=very important). Responses to these two questions were combined to provide an overall assessment of voice (Cronbach’s alpha=0.91) (Mean=3.10, SD=1.11). These questions were drawn from the organisational justice literature, which has highlighted the importance of individual voice in decision-making (Price et al., 2006). ‘Having a say’ in decision-making is a component of procedural justice and has been identified as important in the acceptance of technology (Devine-Wright, 2013).

*Engagement preferences* was measured by asking participants ‘In terms of how you would like to be involved in decision-making, to what extent would you want to be involved in the following: (1=not at all to 5=very much so). I would want to:

- ‘participate in public information sessions about this technology (e.g., town hall meetings)’ (Mean=2.70, SD=1.22)
- ‘access information and/or provide feedback about the technology through social media’ (Mean=3.10, SD=1.24)
- ‘formally contribute to making decisions about the technology (e.g., written submissions to the relevant authority)’ (Mean=2.48, SD=1.24)
- ‘receive results of research on this technology (e.g., a summary report)’ (Mean=3.17, SD=1.23)

These activities were chosen to represent some of the most common options for community engagement, including activities that aimed to inform (i.e., receive results of research – e.g., a summary report), consult (i.e., access information and/or provide feedback through social media), and involve (i.e., formally contribute to making decisions – e.g., written submissions to the relevant authority; participate in public information sessions – e.g., town hall meetings) people in decision-making.

*Personal information needs* were measured by asking participants ‘If information were to be made available, can you please select the top three issues that you would like to hear more about, with your first preference (1) being the most important. I would like to hear more about:

- ‘what the scientific processes and techniques are’ (selected in the top 3 by *n*=328, 28.5%)
- ‘who is funding the research and why’ (selected in the top 3 by *n*=293, 25.5%)
- ‘what the claimed benefits are’ (selected in the top 3 by *n*=235, 20.5%)
- ‘what the possible risks are’ (selected in the top 3 by *n*=695, 60.5%)
- ‘who will benefit and who will bear the risks’ (selected in the top 3 by *n*=336, 29.2%)
- ‘what is being done to regulate and control the technology’ (selected in the top 3 by *n*=595, 51.8%)
- ‘what is being done to deal with the social and ethical issues involved’ (selected in the top 3 by *n*=335, 29.2%)

Around 18% (n=210) indicated that they did not actually need or want to know anything more about the technology. The remaining participants (n=939, 82%) indicated their top 3 information needs as shown above. These information needs were chosen to represent some of the more common issues raised in previous qualitative research on synthetic biology (Bhattachary et al., 2010; Pauwels, 2013).

Analysis

Latent profile analysis was performed to identify the audience segments. Latent profile analysis is a person-centred statistical method for identifying related cases from multivariate continuous data (Howard & Hoffman, 2017; Spurk et al., 2020; Woo et al., 2018). The number and characteristics of the profiles are not predetermined but rather, are identified after the analysis to determine the best fitting model (Fletcher et al., 2012). It aims to identify the smallest number of distinct groups of similar individuals that best represent the patterns in the data, and groups are represented by a categorical latent variable. We used this technique to identify groups of people with similar attitudinal and belief patterns. This type of analysis categorises people into mutually exclusive and exhaustive sub-groups whose members show similarity in their responses on the observed indicators. A series of latent profile analyses were conducted to examine the heterogeneity in beliefs and attitudes relating to the synthetic biology solution. All authors reviewed the results of the latent profile analyses to ensure the chosen profile option was appropriate and meaningful. Analysis of variance (ANOVA) with Bonferroni-adjusted pairwise comparisons was then performed to examine differences in attitudes across the groups. Cohen’s d also was requested to provide an indication of the size of the effects (Cohen’s d=0.2 was a small effect; Cohen’s d=0.5 was a moderate effect; and Cohen’s d=0.8 was considered a large effect). ANOVA also was performed to identify group differences on importance of having a say and involvement in various decision-making opportunities. Logistic regressions were also performed to identify differences among the groups in the information needs selected by participants.

References

Ajzen, I. (1991). The theory of planned behavior. Organizational Behavior and Human Decision Processes, 50(2), 179–211. https://doi.org/10.1016/0749-5978(91)90020-T

Ajzen, I. (2008). Attitudes and the prediction of behavior. In W. D. Crano & Prislin R. (Eds.), Attitudes and attitude change (pp. 289–311). PsychologyPress. https://www.researchgate.net/publication/264156918

Bhattachary, D., Calitz, J. P., & Hunter, A. (2010). Synthetic Biology Dialogue. www.epsrc.ac.uk

Devine-Wright, P. (2013). Explaining “NIMBY” Objections to a Power Line: The Role of Personal, Place Attachment and Project-Related Factors. Environment and Behavior, 45(6), 761–781. https://doi.org/10.1177/0013916512440435

Dunlap, R. E., Van Liere, K. D., Mertig, A. G., & Jones, R. E. (2000). Measuring endorsement of the New Ecological Paradigm: A revised NEP scale. Journal of Social Issues, 56(3), 425-442.

Fletcher, J. M., Marks, A. D. G., & Hine, D. W. (2012). Latent profile analysis of working memory capacity and thinking styles in adults and adolescents. Journal of Research in Personality, 46(1), 40-48. https://doi.org/10.1016/j.jrp.2011.11.003

Howard, M. C., & Hoffman, M. E. (2017). Variable-Centered, Person-Centered, and Person-Specific Approaches. Organizational Research Methods, 21(4), 846-876. https://doi.org/10.1177/1094428117744021

MacDonald, E. A., Balanovic, J., Edwards, E. D., Abrahamse, W., Frame, B., Greenaway, A., Kannemeyer, R., Kirk, N., Medvecky, F., Milfont, T. L., Russell, J. C., & Tompkins, D. M. (2020). Public Opinion Towards Gene Drive as a Pest Control Approach for Biodiversity Conservation and the Association of Underlying Worldviews. Environmental Communication, 14(7), 904-918. https://doi.org/10.1080/17524032.2019.1702568

Pauwels, E. (2013). Public understanding of synthetic biology. In BioScience, 63(2), 79–89. <https://doi.org/10.1525/bio.2013.63.2.4>

Price, K. H., Lavelle, J. J., Henley, A. B., Cocchiara, F. K., & Buchanan, F. R. (2006). Judging the fairness of voice-based participation across multiple and interrelated stages of decision making. Organizational Behavior and Human Decision Processes, 99(2), 212–226. https://doi.org/10.1016/j.obhdp.2005.10.005

Priester, J. R., & Petty, R. E. (1996). The gradual threshold model of ambivalence: Relating the postiive and negative bases of attitudes to subjective ambivalence. Journal of Personality and Social Psychology, 71, 431-449.

Rogers, E. (2003). Diffusion of Innovations Theory (5th ed.). FreePress.

Rogers R. (1975). A Protection Motivation Theory of Fear Appeals and Attitude Change. The Journal of Psychology, 91(1), 93–114. https://doi.org/10.1080/00223980.1975.9915803

Rogers R. (1983). Cognitive and physiological processes in attitude change: A revised theory of protection motivation. In J. Cacioppo & R. Petty (Eds.), Social Psychophysiology (pp. 153–176). GuilfordPress.

Siegrist, M., & Cvetkovich, G. (2000). Perception of hazards: The role of social trust and knowledge. Risk Analysis, 20(5), 713–720. https://doi.org/10.1111/0272-4332.205064

Spurk, D., Hirschi, A., Wang, M., Valero, D., & Kauffeld, S. (2020). Latent profile analysis: A review and “how to” guide of its application within vocational behavior research. Journal of Vocational Behavior, 120. https://doi.org/10.1016/j.jvb.2020.103445

Watson, D., Clark, L. A., & Tellegen, A. (1988). Development and Validation of Brief Measures of Positive and Negative Affect: The PANAS Scales. Journal of Personality and Social Psychology, 54(6), 1063-1070.

Woo, S. E., Jebb, A. T., Tay, L., & Parrigon, S. (2018). Putting the “Person” in the Center. Organizational Research Methods, 21(4), 814-845. https://doi.org/10.1177/1094428117752467

Wurpts, I. C., & Geiser, C. (2014). Is adding more indicators to a latent class analysis beneficial or detrimental? Results of a Monte-Carlo study. Frontiers in Psychology, 5, 920. https://doi.org/10.3389/fpsyg.2014.00920

Zhang, A., Measham, T. G., & Moffat, K. (2018). Preconditions for social licence: The importance of information in initial engagement. Journal of Cleaner Production, 172, 1559-1566. https://doi.org/10.1016/j.jclepro.2017.10.323
